# Nanoparticles as Smart Carriers for Enhanced Cancer Immunotherapy

**DOI:** 10.3389/fchem.2020.597806

**Published:** 2020-12-21

**Authors:** Neelam Thakur, Saloni Thakur, Sharmistha Chatterjee, Joydeep Das, Parames C. Sil

**Affiliations:** ^1^Himalayan Centre for Excellence in Nanotechnology, Shoolini University, Solan, India; ^2^School of Advanced Chemical Sciences, Faculty of Basic Sciences, Shoolini University, Solan, India; ^3^Faculty of Applied Sciences and Biotechnology, Shoolini University, Solan, India; ^4^Division of Molecular Medicine, Bose Institute, Kolkata, India

**Keywords:** adjuvants, antigens, cancer, immunostimulatory nanoparticles, immunotherapy, theranostics

## Abstract

Cancer immunotherapy has emerged as a promising strategy for the treatment of many forms of cancer by stimulating body's own immune system. This therapy not only eradicates tumor cells by inducing strong anti-tumor immune response but also prevent their recurrence. The clinical cancer immunotherapy faces some insurmountable challenges including high immune-mediated toxicity, lack of effective and targeted delivery of cancer antigens to immune cells and off-target side effects. However, nanotechnology offers some solutions to overcome those limitations, and thus can potentiate the efficacy of immunotherapy. This review focuses on the advancement of nanoparticle-mediated delivery of immunostimulating agents for efficient cancer immunotherapy. Here we have outlined the use of the immunostimulatory nanoparticles as a smart carrier for effective delivery of cancer antigens and adjuvants, type of interactions between nanoparticles and the antigen/adjuvant as well as the factors controlling the interaction between nanoparticles and the receptors on antigen presenting cells. Besides, the role of nanoparticles in targeting/activating immune cells and modulating the immunosuppressive tumor microenvironment has also been discussed extensively. Finally, we have summarized some theranostic applications of the immunomodulatory nanomaterials in treating cancers based on the earlier published reports.

## Introduction

Nowadays immunotherapy has emerged as a promising and innovative strategy which is widely used for the treatment of various types of diseases by modulating the host's immune system. It may be classified as immunosuppressive or immunostimulatory therapy depending on whether it suppresses or activates host's immune response (Singh and Bhaskar, 2014). Immunosuppressive therapy refers to the down-regulation of the immune response which helps in the treatment of various types of autoimmune diseases like type 1 diabetes, obesity, atherosclerosis, and rheumatoid arthritis. On the other hand, immunostimulatory therapy refers to that which activates immune response, thus helping in the treatment of cancer and other infectious diseases (Feng et al., 2019).

In recent years, immunomodulatory approach has gained significant interest in the field of cancer therapy by stimulating the host's immune system to fight against this disease due to its milder side effects as compared with the traditional therapies such as chemotherapy, radiotherapy, and surgery (Yang et al., 2020). Our body's immune system can distinguish cancer cells from normal cells by recognizing tumor antigens which are of two types; (i) tumor-specific antigens (TSAs), i.e., specific molecules that are exclusively produced from cancer cells, like PSA or Prostrate-specific antigens (more specific for cancer recognition and activate host immune response) and (ii) tumor-associated antigens (TAAs), i.e., specific molecules that are produced both from normal cells and cancer cells, like CEA, or Carcinoembryonic antigen (which are produced in much greater quantities from cancer cells, but do not induce host immune response) (Koury et al., 2018). However, the immunosuppressive nature of tumor microenvironment (TME) inhibits the ability of the host immune system to function effectively against tumor cells (Musetti and Huang, 2018). The activation of the host's immunity can be done by various approaches such as, introduction of various cancer vaccines, monoclonal antibodies, immune checkpoint blockers, and cell-based therapies which have been proven to be very effective in many patients (Ventola, 2017). Cancer immunotherapy not only treats cancer by inducing strong anti-tumor immune response but also controls metastasis as well as prevents its recurrence; hence representing a major advantage over traditional cancer treatments (Hodi et al., 2010; Kroemer and Zitvogel, 2018). However, some limitations are also associated with the existing cancer immunotherapy such as induction of destructive auto-immunity (Phan et al., 2003) and lack of effective delivery of cancer antigens to immune cells (Buabeid et al., 2020). Besides, the immunosuppressive TME itself attenuates the efficacy of cancer immunotherapy (Zou, 2005; Munn and Bronte, 2016).

**Figure 1:**
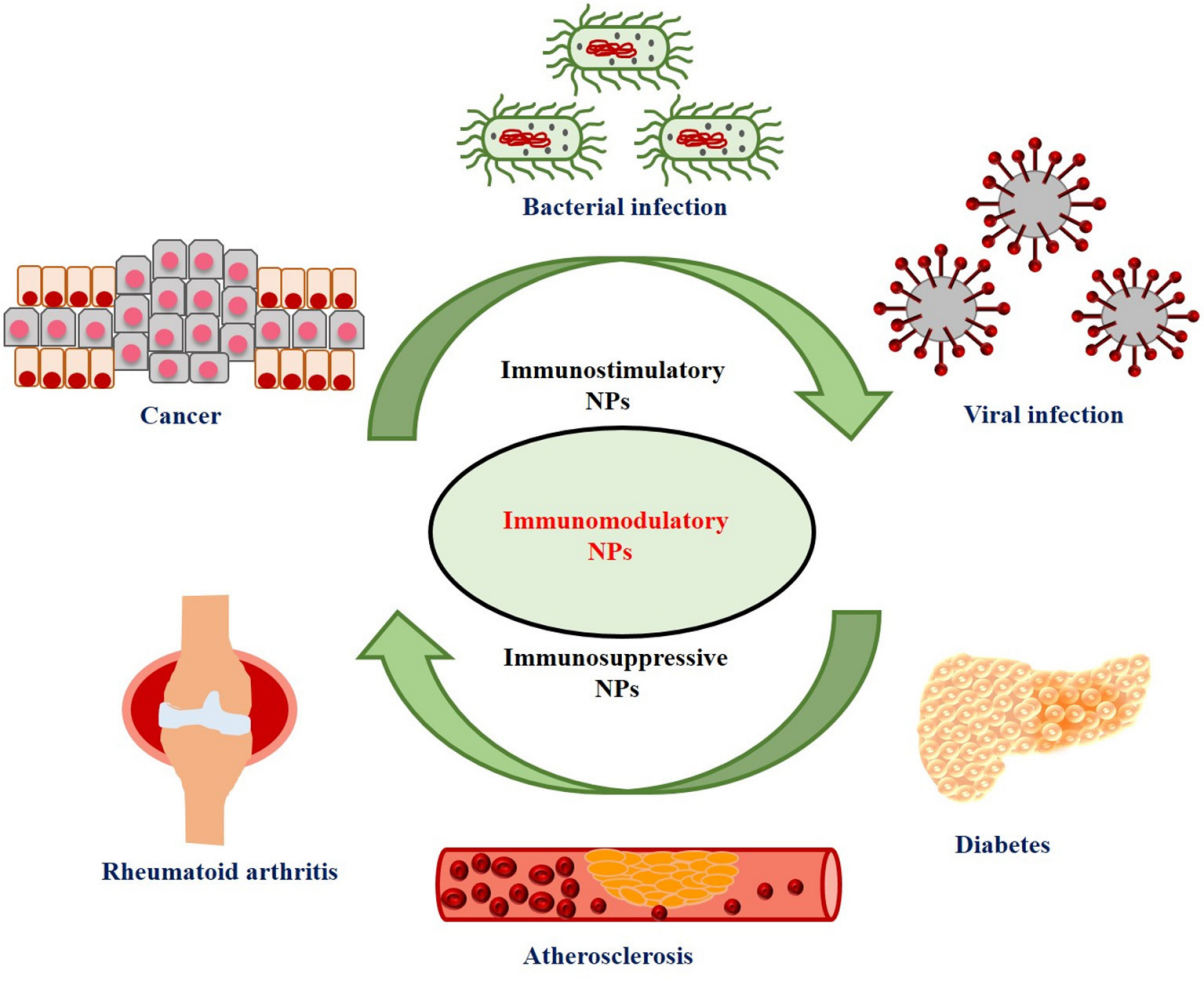
Applications of immunostimulatory and immunosuppressive nanoparticles (NPs) for the treatment of various diseases.

Nanotechnology offers the opportunity to overcome these shortcomings of traditional cancer immunotherapy and thus enhances its efficacy (Jo et al., 2017). Nanoparticles (NPs) have several unique properties such as small particle size, adjustable shape, high cell penetration ability, and enhanced/improved magnetic, electronic, mechanical, and optical properties as compared to its bulk structures (Fard et al., 2015). Due to these remarkable properties, NPs are widely used in various biomedical applications (Das et al., 2016, Das et al., 2017; Sharma and Das, 2019; Thakur et al., 2019; Tejwan et al., 2020). NPs show profound immunomodulatory effects (immunosuppressive or immunostimulating) and therefore, have been used for the treatment of various types of diseases (Jiao et al., 2014). Immunosuppressive NPs are used for the treatment of inflammatory and autoimmune diseases; whereas, immunostimulatory NPs are used for the treatment of cancer and some other infectious diseases (Feng et al., 2019). The applications of immunostimulatory and immunosuppressive NPs for the treatment of various diseases have been shown in Figure 1. Besides, NPs have also been extensively used as carriers or delivery vehicles in cancer immunotherapy due to its numerous advantageous properties, such as (1) simultaneous delivery of antigens and adjuvants to the same antigen presenting cells (APCs); (2) protection of bioactive cargo molecules from enzymes-induced degradation; (3) effective delivery toward specific cells or tissues by functionalization with targeting ligands; (4) promoting the development of long term memory response; and (5) intrinsic adjuvant properties of some nanocarriers avoids the need for additional adjuvants (Fontana et al., 2018; Feng et al., 2019). Hence, NPs have emerged as a promising carrier for immunostimulating agents (antigens/adjuvants) and thus plays an important role in cancer immunotherapy. Nanoparticles can also be engineered to act as mediators of tumor destruction via methods like photodynamic therapy (PDT) (Canti et al., 2010; Kim D. et al., 2020), photothermal therapy (PTT) (Yang et al., 2019; Xu and Liang, 2020), or by acting as radiosensitizers (Boateng and Ngwa, 2019; Jin and Zhao, 2020), to increase cancer antigen release, thus activating the body's immune system.

In the present review article, we have largely focussed on the use of nanoparticles as antigen/immunostimulant delivery vehicles, leaving room for discussion about their use in photo and radio-based immunotherapy for a later literature review. Here we would like to first introduce some immunostimulatory NPs that have widely been used in immunotherapy. Then we have extensively discussed about the role of NPs as smart carriers of antigen/adjuvants for cancer immunotherapy. After that, we would like to discuss about the types of interactions between NPs and antigen/adjuvant as well as various factors that control the interaction of NPs with immune cells. In addition to this, several strategies for the NPs to activate the immune cells like dendritic cells (DCs), T-cells and natural killer cells (NK cells) as well as the immunomodulating effects of the NPs in the TME would also be discussed in details. Finally, the applications of NPs in cancer theranostics would be reviewed.

## Types of Nanoparticles for Immunostimulation

Immunomodulators are natural or synthetic molecules that can normalize or modulate our body's immune system. These substances can further be divided into two categories: immunosuppressants (which suppress our immune system) and immunostimulants (which stimulate our immune system) (Patil et al., 2012). The immunostimulatory effects of engineered NPs that have been and are being widely used in cancer immunotherapy are summarized in Table 1 and discussed in the subsequent sections.

**Table 1:** Nanoparticle-based immunostimulatory effect for cancer therapy.

**S. No**	**Nanoparticles**	**Payload**	**Outcomes**	**References**
1.	Chitosan	Tumor cell lysates	Enhanced the efficacy of antitumor immune response	Shi et al., 2017
2.	Chitosan	Mucin1 (MUC1) glycopeptide antigens	Improved the immunogenicity of peptide epitope, produced significant IgG antibodies and elicited strong antigen specific immune response	Chen et al., 2017
3.	PLGA	TLR 7 agonist	Enhanced uptake of nanovaccine by DCs which further trigger anti-tumor immune response	Yang et al., 2018
4.	PLGA	TLR 7/8 agonist	Enhanced antigen specific immune response	Kim et al., 2018
5.	PLGA	Poly (I:C), Resiquimod and MIP 3α	Enhanced the efficiency of cancer vaccine	Da Silva et al., 2019
6.	Liposomes	OVA	Induced antigen-specific immunity	Yuba et al., 2014
7.	Liposomes	CpG-ODN and 3,5-didodecyloxybenzamidine	Induced the production of cytokines from DCs, expression of co-stimulatory molecules and enhanced antigen- specific immune response	Yoshizaki et al., 2017
8.	Liposomes	SLPs antigens	Induced antigen specific CD8^+^ T cells mediated immune response	Heuts et al., 2018
9.	GNPs	-	Induced immune cell response by activating macrophages	Fallarini et al., 2013
10.	GNPs	CpG-ODN and BSA antigen	Detection of higher anti- BSA antibodies in blood serum of mice immunized with BSA–GNP and CpG–GNP conjugates	Dykman et al., 2018
11.	GNPs	-	Modulated TME and inhibit tumor growth	Melamed et al., 2016
12.	GNPs	-	Inhibited tumor growth by reprogramming pancreatic TME	Saha et al., 2016
13.	GNPs	CpG- ODNs	Enhanced cancer associated immunostimulatory activity as compared to free CpG-ODNs	Luo J. et al., 2019
14.	MWCNTs	Cancer testis antigen (NY-ESO-1) and CpG- ODNs	Induced strong CD4^+^ T as well as CD8^+^ T cell-mediated immune response against NY-ESO-1	Faria et al., 2014
15.	MWCNTs	OVA, CpG-ODN, and anti- CD 40 Ig	Enhanced OVA specific T cell responses and inhibited the growth of OVA–expressing B16F10 melanoma cells in subcutaneous or lung pseudo-metastatic tumor models	Hassan et al., 2016
16.	MWCNTs	CpG- ODNs	Induced strong humoral and cellular immune response	Xia et al., 2018
17.	MWCNTs	OVA	Induced strong anti-tumor immune response	Dong et al., 2019
18.	MSNs	OVA	Elicited both antibody and cell -mediated immune responses	Mahony et al., 2013
19.	Hollow MSNs	Doxorubicin (DOX), all-trans retinoic acid (ATRA), and interleukin-2 (IL-2)	Regulated TME and enhanced anti-tumor effect	Kong et al., 2017
20.	Mesoporous organosilica NPs	OVA and unmethylated CpG-ODNs	Induced strong CD8^+^ T cells response and enhanced anti-tumor activity	Lu et al., 2018
21.	MSNs	CpG-ODNs	Increased the secretion of IL-12 and TNF-α as compared to free CpG-ODNs	Ong et al., 2019
22.	MSNs	OVA	Increased cytotoxic CD8^+^ T cells which significantly suppressed tumor growth and enhanced the survival rate of C57BL/6 mice	Lee et al., 2020
23.	Magnetic NPs	Anti-tumorigenic cytokine, IFN-γ	Efficient accumulation of NPs at the tumor site which stimulate anti-tumor immune response and significantly reduced tumor size	Mejías et al., 2011
24.	Magnetic NPs	-	Enhanced T cell activation and stimulates anti-tumor activity	Perica et al., 2014
25.	Magnetic NPs	OVA, poly (I:C) and imiquimod (R837)	Significantly enhanced anti-tumor immune response	Gondan et al., 2018
26.	Iron oxide (Fe_3_O_4_) NPs	OVA	Induced a strong adaptive immune response by activating DCs and macrophages *in vitro* as well as inhibited tumor growth and prevented tumor formation *in vivo*	Luo L. et al., 2019
27.	Magnetic NPs	OVA	Induced a strong CD8^+^ as well as CD4^+^ T cell mediated immune response	Lee et al., 2019
28.	Protein NPs	Peptide epitope and CpG-ODNs	Enhanced CD8^+^ T cell and antigen cross-presentation	Molino et al., 2013
29.	Protein NPs	Melanoma-associated gp100 epitope and CpG-ODN	Significant increase in antigen-specific anti-tumor immune response	Molino et al., 2016
30.	Protein NPs	NY-ESO-1, MAGE A3, and CpG-ODN	Induced a significant antigen- specific cell- mediated immune response	Neek et al., 2018
31.	Micelles	Trp2 and CpG- ODNs	Triggered antigen- specific cytotoxic CD8^+^ T cell mediated immunity and induced a potent antitumor immune response in tumor bearing mice	Zeng et al., 2017
32.	Micelles	Trp2 and CpG- ODNs	Trp2/PHM10/CpG nanoformulation significantly generated a strong CD8^+^ T cell activity as well as enhanced the anticancer efficacy	Li et al., 2017
33.	Micelles	OVA and CL264 agonist	Elicited a potent antigen specific humoral and cellular immune response	Li C. et al., 2018
34.	VLPs	-	Induced a significant antitumor immune response in tumor bearing mice by modulating TME	Lizotte et al., 2016
35.	VLPs	-	Generated a strong antitumor immune response by modulating TME which subsequently results in immunological regression of glioma	Kerstetter-Fogle et al., 2019
36.	VLPs	CH401 peptide	Induced a strong anti HER- 2 immune response that delayed tumor growth and prolonged the survival rate in immunized mice	Shukla et al., 2019
37.	VLPs	CpG-ODNs	Enhanced the efficacy of CpG-ODNs against tumor and induced a potent antitumor response	Cai et al., 2020
38.	Clec9A- TNE	OVA and HPV16 antigen E6/E7	Induction of a potent antigen specific immunity	Zeng et al., 2018
39.	NEs	TLR7/8 agonists, OVA, and long peptide of E7 antigen	Enhanced the efficacy of cancer immunotherapy by activating DCs, T cells as well as by reprogramming TME	Kim et al., 2019
40.	Nanogels	OVA	Enhanced the maturation of DCs and promoted lysosomal rupture which further increased the level of ROS and facilitated antigen presentation thus, evoked a strong anticancer immune response	Wang et al., 2016
41.	Nanogels	OVA and poly (I:C)	Elicited a potent OVA specific immune response against melanoma	Li D. et al., 2016
42.	Nanogels	OVA	Effective delivery of OVA to DCs which further evoked a strong antigen specific adaptive immunity	Miura et al., 2019
43.	Dendrimers	OVA and CpG-ODNs	Induced a significant higher T-cell mediated immune response	Xu et al., 2019
44.	Dendrimers	CpG-ODNs	Effective delivery of CpG-ODNs into DCs elicited adaptive cellular immune response	Chen et al., 2020

### Polymeric Nanoparticles

Polymeric NPs are the most widely used immunostimulatory NPs as they exhibit excellent biocompatibility, biodegradability, chemical stability, water solubility, and high capacity to load immune-related components (Li S. et al., 2018). The commonly used polymeric NPs in cancer immunotherapy are poly (D, L-lactic-coglycolic acid) (PLGA), poly (g-glutamic acid) (PGA), poly (D,L-lactide-co-glycolide) (PLG), poly (ethylene glycol) (PEG), poly ethylenimine (PEI), and chitosan NPs (Zhao et al., 2014). These NPs have extensively been employed as an effective immunostimulatory adjuvant in vaccination (Chen et al., 2017; Shi et al., 2017; Yang et al., 2018). For example, Kim et al. (2018) showed that encapsulation of toll-like receptor 7/8 (TLR 7/8) agonist within PLGA NPs significantly increased the co-stimulatory molecule expression (CD40, CD80 and CD86) as compared with free agonist *via* the activation of bone marrow-derived dendritic cells (BMDCs). Besides, subcutaneous administration of the nanoformulation leads to its migration to the draining lymph nodes, where it subsequently activates DCs as well as CD8^+^ T cells (cytotoxic T cells), resulting in increased anticancer response in bladder, melanoma and renal carcinoma models, thereby proving the role of PLGA NPs as potent immunostimulatory adjuvants for cancer immunotherapy. In a recent study, Da Silva et al. (2019) used PLGA NPs for the co-delivery of two TLR agonists (polyinosinic: polycytidylic acid [poly (I:C)] and Resiquimod) in combination with a chemokine, MIP 3α (Macrophage Inflammatory Protein-3 alpha) to significantly enhance the therapeutic efficacy of cancer vaccines in tumor bearing mice. PLGA NPs-mediated co-delivery of these immune modulators significantly altered the lymphoid and myeloid cell populations in the tumor and tumor-draining lymph node. Besides, such nanovaccines improved the long- term survival of tumor bearing mice to 75–100% as well as nearly doubled the progression- free survival time of the mice.

### Liposomes

Liposomes have also emerged as an important NPs and used as a delivery vehicle for drugs, genes, as well as vaccines (Banerjee, 2001). Several liposomal formulations such as 1,2-dioleoyl-3-trimethylammonium-propane (DOTAP), 3β- (N- [N',N'-dimethyl aminoethane] - carbamoyl) cholesterol (DC-Chol), and dimethyl diocta decylammonium (DDA) (Klinguer-Hamour et al., 2002; Christensen et al., 2011) have been employed for effective delivery of antigens to APCs and also served as vaccine adjuvants, thereby enhancing the antigen-specific immune responses (Smith Korsholm et al., 2007; Zamani et al., 2018). Yuba et al. (2014) showed that pH-responsive dextran-modified liposomes were efficiently taken up by DCs and delivered the entrapped ovalbumin (OVA) into the cytosol. Besides, subcutaneous administration of the nanoformulation resulted in increased antigen-specific immune responses and suppression of tumor growth in E.G7-OVA tumor bearing mice. In another study, Yoshizaki et al. (2017) reported that inclusion of cytosine-phosphate-guanine oligodeoxynucleotides (CpG-ODNs, a TLR9 agonist) and 3,5-didodecyloxybenzamidine (adjuvant) into pH-responsive polymer-modified liposomes promoted the expression of co-stimulatory molecules and production of cytokine from DCs; thus, resulted in enhanced antigen-specific immunity. These findings revealed the profound application of liposomes as antigen carriers and adjuvants in cancer immunotherapy. Besides, Heuts et al. (2018) reported that cationic liposomes could efficiently deliver synthetic long peptides (SLPs) antigens to DCs and promoted antigen cross- presentation, thereby resulting in the activation of CD8^+^ cytotoxic T-cells. Hence, liposomes can be considered as an efficient delivery system for peptide-based cancer vaccines.

### Gold Nanoparticles

Gold nanoparticles (GNPs) are also used in immunotherapy due to their low cytotoxicity, tunable surface chemistry, and easily controllable shape and size (Zhou et al., 2016). GNPs are an important class of immunostimulatory NPs which show its response by activating macrophages and their subsequent differentiation into dendritic-like cells, leading to T-cell proliferation and cytokine release (Fallarini et al., 2013). GNPs were also found to be useful as an adjuvant for antibody production in mice (Dykman et al., 2018), and its immunogenic property was further increased when used in combination with another immunostimulant, CpG-ODNs. Moreover, GNPs can inhibit tumor growth by modulating TME (Melamed et al., 2016; Saha et al., 2016). Recently, Luo J. et al. (2019) conjugated thiolated CpG-ODNs on the surface of hollow GNPs and observed a significantly higher cellular uptake of the nanoconjugate by immune cells and enhanced immune stimulatory activity as compared to free CpG-ODNs. CpG-hollow GNPs increased the secretion of TNF α from RAW264.7 cells by ~15 fold as compared to CpG-ODNs alone.

### Carbon Nanotubes

Carbon nanotubes (CNTs) have also been known to induce immunostimulatory effects in both *in vitro* and *in vivo* systems. Faria et al. (2014) used oxidized multiwalled carbon nanotubes (MWCNT) for the delivery of cancer-testis antigen (NY-ESO-1, New York esophageal squamous cell carcinoma-1) and CpG-ODNs. They showed that the nanoplatforms were rapidly uptaken by DCs and induced a strong CD4^+^ (Helper) T-cell and CD8^+^ (cytotoxic) T cell-mediated immune response, resulting in delayed tumor growth in B16F10 melanoma tumor bearing mice. Later, Hassan et al. (2016) checked the co-delivery of an antigen, OVA along with CpG-ODN and anti-CD40 Ig as immunoadjuvants using MWCNTs. They showed that utilization of such MWCNTs as delivery vehicles significantly potentiated OVA-specific T cell responses and inhibited the growth of OVA–expressing B16F10 melanoma cells in subcutaneous or lung pseudo-metastatic tumor models. Xia et al. (2018) synthesized MWCNTs conjugated with H3R6 polypeptide for the effective and targeted delivery of CpG-ODNs into prostate cancer. Their *in vitro* and *in vivo* studies demonstrated strong humoral and cellular immune stimulatory abilities of the nanocomposites as evident by the increased expression of TNF-α, IL-6, CD4^+^ T, and CD8^+^ T-cells. Recently, Dong et al. (2019) used mannose- modified MWCNTs as an efficient nanovector for the delivery of a tumor antigen, OVA into DCs. They observed that these nanovectors were rapidly taken up by DCs and further enhanced DCs maturation and cytokine secretion to trigger strong anti-tumor immune response. All these studies clearly demonstrated that CNTs can be used as an immunostimulatory agent as well as a delivery vehicle for antigen and adjuvants for cancer immunotherapy.

### Silica Nanoparticles

Silica NPs are mostly biocompatible and widely used in various biomedical applications, like bioimaging (Ow et al., 2005), tumor targeting (Benezra et al., 2011), and drug/vaccine delivery (Chattopadhyay et al., 2017). Mahony et al. (2013) investigated the role of MSN (mesoporous silica nanoparticle) as an efficient antigen delivery vehicle as well as its self-adjuvant effect by immunizing mice with OVA -loaded amino-functionalized MSN. They showed that such nanovaccine elicited both humoral and cell-mediated immune responses without causing any cytotoxic effect in mice at very low antigen doses, thus demonstrating their self-adjuvant potential and biocompatibility in vaccine delivery applications. Besides, Kong et al. (2017) demonstrated the feasibility of using biodegradable hollow MSN for regulating TME and enhancing antitumor efficacy. In another study, Lu et al. (2018) reported the effective role of glutathione depletion dendritic mesoporous organosilica nanoparticles (GDMON) as a novel self-adjuvant as well as a co-delivery nanocarrier for enhanced cancer immunotherapy. GDMON effectively co-delivered OVA and unmethylated CpG-ODNs into APCs and induced endosomal escape. Moreover, these NPs decreased the intracellular glutathione (GSH) level and increased the ROS (reactive oxygen species) level which further induced strong cytotoxic T cell (CD8^+^ T cell) response as well as enhanced antitumor activity. In another study, Ong et al. (2019) decorated extra-large porous MSNs with small GNPs for effective delivery of CpG-ODNs into DCs. They showed that delivery of CpG-ODNs using such nanoplatform enhanced the expression of co-stimulatory molecules as well as increased the secretion of pro-inflammatory cytokines (IL-12 and TNF-α) as compared to soluble CpG-ODNs *in vitro*. Besides, the *in vivo* study also demonstrated a significant tumor growth inhibition and enhanced survival rate in tumor-bearing mice treated with CpG-ODN loaded NPs as compared to soluble CpG-ODNs. Recently, Lee et al. (2020) used PEI-coated hollow MSNs with extra-large mesopores to encapsulate OVA for the effective activation of DCs and induction of antigen-specific immune response. Besides, the *in vivo* study also demonstrated that such nanovaccines enhanced the population of antigen-specific CD8^+^ T cells, which significantly suppressed tumor growth as well as improved the survival rate of tumor-bearing mice.

### Magnetic Nanoparticles

Currently, magnetic NPs have been widely used in theranostic domain due to their magnetic resonance imaging (MRI) properties (Sau et al., 2018). Among these NPs, superparamagnetic iron oxide nanoparticles (SPIONs) are attracting much interest of many researchers for cancer theranostic applications (Gobbo et al., 2015). These NPs are often coated with a layer of biocompatible materials in order to reduce their aggregation. In a study conducted by Mejías et al. (2011), it has been observed that dimercaptosuccinic acid (DMSA) coated (SPIONs) effectively delivered an anti-tumorigenic cytokine, IFN-γ, at the tumor site by using an external magnetic field. The results of this study also showed an efficient accumulation of the NPs and cytokine release at the tumor site, leading to enhanced T-cell populations, macrophage infiltration, anti-angiogenesis as well as suppression of tumor growth. Besides, magnetic NPs coated with dextran and functionalized with T-cell activating proteins have been found to enhance T-cell activation by using an external magnetic field and further inhibit tumor growth (Perica et al., 2014). In another study, Gondan et al. (2018) loaded two TLR agonists, poly (I:C) and imiquimod (R837) in combination with a model antigen, OVA, onto micellar zinc-doped iron oxide magnetic NPs and observed a synergistic activation of anti-tumor immune response and direct killing effect on cancer cells. Recently, Luo L. et al. (2019) reported the use of OVA-loaded ultrasmall iron oxide nanocomposites for efficient DCs maturation and T cell activation. In addition, these nanocomposites also activated macrophages, thus promoted a significant adaptive immune response against tumor. The *in vivo* results demonstrated that these nanocomposites could inhibit the subcutaneous and metastatic B16-OVA tumor growth as well as prevent the tumor formation. In the same year, Lee et al. (2019) showed that OVA conjugated and silica coated magnetic NPs were efficiently taken up by DCs and activated antigen specific CD4^+^ T helper type 1 (Th1) cell responses as well as induced antigen-specific CD8^+^ cytotoxic T (T_c_) cell immune responses. Besides, these nanoformulations significantly inhibited tumor growth in EG7-OVA tumor bearing mice.

### Protein Nanoparticles

Protein NPs have also been used as effective vaccine platforms for delivering tumor antigens and adjuvants to induce a strong anti-tumor immune response. For example, a study performed by Molino et al. (2013) demonstrated that biomimetic protein NPs could effectively co-deliver peptide epitopes and CpG-ODN activator to DCs, resulting in increased and prolonged CD8^+^ T cell activation as well as enhanced antigen cross-presentation. In another study, it has been observed that simultaneous treatment with melanoma-associated gp100 epitope and CpG-ODN using viral mimicking protein NPs significantly increased CD8^+^ T cell proliferation and IFN-γ secretion (Molino et al., 2016). Furthermore, immunization of mice with such multifunctional NPs delayed their tumor growth onset by ~5.5 days and increased surviving time of the animal by ~ 40%. In 2018, Neek et al. (2018) reported the simultaneous delivery of two human cancer-testis antigens, HLA-A2 restricted epitopes of MAGE-A3 (Melanoma antigen gene family-A3) and NY-ESO-1 using the E2 subunit assembly of pyruvate dehydrogenase (E2 nanoparticle). They observed that simultaneous delivery of NY-ESO-1 epitope and CpG-ODN using E2 protein NPs resulted in 25- fold higher IFN-γ secretion and 15- fold higher cell lysis activity in HLA- A2 transgenic mice model. Immunization of mice with MAGE-A3 epitope and CpG-ODN using E2 NPs resulted in an increase in antigen-specific IFN-γ secretion by 6- fold and increased in cell lysis activity by 9- fold. However, the combined delivery of NYESO1-CpG- E2 and MAGEA3- CpG- E2 nanoformulations resulted in an additive effect in favor of IFN-γ secretion and cell lysis activity.

### Micelles

Micelles have also been used as an effective nanocarrier of antigen/adjuvant for enhancing the potency of cancer vaccines. For example, in a study Zeng et al. (2017) used polymeric hybrid micelles (PHMs) for encapsulating melanoma antigen peptides (Trp2) and TLR- 9 agonists (CpG-ODN). They observed that the nanovaccine could effectively target the proximal lymph node and promoted the internalization of antigen/adjuvant into DCs. This co-delivery system further triggered antigen-specific CD8^+^ T cell immune responses and induced strong anti-tumor effect in lung metastatic melanoma model. In another study, Li et al. (2017) designed different formulations of polymeric hybrid micelles (PHMs) using cationic polycaprolactone-polyethylenimine (PCL-PEI) and polycaprolactone–polyethylene glycol (PCL–PEG) for the effective co-delivery of Trp2 peptide antigen and CpG-ODN adjuvant. They observed that the PHMs having 10 % (w/w) PCL-PEI (Trp2/PHM10/CpG) were effectively taken up by DCs and induced stronger antigen-specific CD8^+^ T cell immune responses as well as antitumor efficacy as compared to the mixture of free Trp2 and CpG or Trp2/PHM0/CpG without PCL-PEI. Later in 2018, Li C. et al. (2018) synthesized carboxylated polymeric mixed micelles for the effective co-delivery of OVA and TLR-7 agonist (CL264) to DCs in lymph nodes. They demonstrated that such nanovaccines further induced DC maturation, cytokine secretion and enhanced antigen cross-presentation which results in the induction of strong antigen-specific immune response. Moreover, immunization with such nanovaccine could significantly inhibit tumor growth in C57BL/6 mice.

### Virus Like Particles

Virus like particles (VLPs) has also attracted significant interest as an ideal nanovaccine platform that has been used to enhance the efficacy of cancer immunotherapy. A study by Lizotte et al. (2016) demonstrated that inhalation of self-assembled VLPs derived from cowpea mosaic virus (CPMV) significantly reduced B16F10 lung melanomas and induced strong systemic antitumor immune response in C57BL/6J mice by activating neutrophils in TME. Recently, Kerstetter-Fogle et al. (2019) also reported that CPMV VLPs induced a potent antitumor immune response against intracranial glioma. Shukla et al. (2019) developed CPMV VLPs based cancer vaccines conjugated with the HER-2 derived antigenic CH401 peptide for generating a sustained and potent anti HER-2 immune response in HER2^+^ mice cancer models. They also showed that such nanovaccines delayed the tumor growth and metastasis as well as prolonged survival in mice. In 2020, Cai et al. (2020) encapsulated CpG-ODNs into VLPs derived from Cowpea chlorotic mottle virus (CCMV) for targeted delivery of CpG-ODNs to tumor-associated macrophages (TAMs) in TME. They further demonstrated that CpG-ODNs loaded VLPs were efficiently taken up by TAMs in TME and enhanced their phagocytic activity as well as induced more effective antitumor activity as compared to free CpG-ODNs both *in vitro* and *in vivo*.

### Nanoemulsions

Nanoemulsions (NEs) are used as adjuvants or as delivery vehicles of antigen/adjuvant for inducing strong antitumor immune response. For example, Zeng et al. (2018) developed C- type lectin receptor (Clec9A) functionalized tailored nanoemulsions (Clec9A- TNE) as a self-adjuvating nanosystem for antigen-specific immunotherapy. They further encapsulated OVA into Clec9A-TNE and observed that such nanovaccine effectively targeted and activated cross-presenting DCs and induced antigen-specific CD4^+^ and CD8^+^ T cell proliferation, as well as antibody and CD8^+^ T cell immune responses. They also loaded oncogenic human papillomavirus 16 (HPV16) antigen, E6/E7 to Clec9A- TNE and demonstrated that HPV16 E6/E7- Clec9A- TNE significantly inhibited tumor growth, stimulated potent antigen specific IFN-γ responses and enhanced the survival of the mice. Recently, Kim et al. (2019) reported that TLR7/8 agonists-loaded NEs along with tumor antigens effectively induced antitumor immune response by activating DCs, T-cells and by modulating immunosuppressive TME without systemic toxicity. Further, they also demonstrated that the combination of NE (TLR 7/8) treatment along with Immune checkpoint blockade therapy (ICBT) induced a synergistic antitumor immune response in B16F10-OVA melanoma and TC-1 cervical tumor model.

### Nanogels

Nanogels have also emerged as an effective antigen or protein delivery system in the field of cancer immunotherapy. Wang et al. (2016) developed amphiphilic pH- sensitive galactosyl dextran-retinal (GDR) nanogels as a promising self- adjuvanting antigen delivery system for targeted delivery of OVA into DCs. They demonstrated that such nanovaccines (GDR/OVA) significantly enhanced DC maturation, antigen uptake by DCs and cytosolic antigen release. GDR nanogels also triggered lysosomal rupture in DCs and facilitated the production of ROS which further promoted antigen cross-presentation by DCs and generated strong antitumor immune response. Li D. et al. (2016) also conjugated OVA to cationic dextran nanogels via disulphide bond which enabled intracellular release of OVA in reductive environment. They observed that such OVA-nanogels were efficiently uptaken by DCs and promoted their maturation. Moreover, in combination with an adjuvant, poly (I:C), these OVA- nanogels significantly induced strong antitumor responses against melanoma *in vivo*. Recently, Miura et al. (2019) developed carboxyl group modified cholesterol-bearing pullulan self-assembly nanogels for OVA delivery into DCs for the induction of significant adaptive immune response.

### Dendrimers

Dendrimer-based nanovaccines have also been used to stimulate the immune system for the treatment of various types of cancer. In a study, Guanidinobenzoic acid (DGBA) modified polyamidoamine dendrimers were used as an effective nanocarrier for the co-delivery of OVA and CpG-ODN (Xu et al., 2019). The combined nanovaccine (DGBA- OVA- CpG) has been efficiently taken up by DCs, promoted DCs maturation and lysosomal escape which further facilitated antigen cross-presentation by DCs. Such nanovaccine significantly induced CD8^+^ T cell immune responses and conferred significant prophylactic efficacy against B16- OVA melanoma. Moreover, in combination with ICBT, such nanovaccine treatment showed synergistic CD8^+^ T cell immune responses against B16- OVA melanoma. Recently, Chen et al. (2020) developed methoxy polyethylene glycol decorated dendrimer-entrapped GNPs (PEG-Au DENPs) for effective delivery of CpG-ODN to DCs. It has been observed that CpG loaded PEG-Au DENPs were efficiently uptaken by DCs and promoted DC maturation which further activated T-cells to trigger an adaptive cellular antitumor immune response.

## Strategies for Optimizing Immunostimulatory Nanoparticles

In cancer immunotherapy, NPs play a vital role in activating host's immune system on the basis of three different strategies: (1) delivering antigens and adjuvants, (2) delivering antigens and acting as a self-adjuvants, and (3) delivering or acting as adjuvants and inducing immunogenic cancer cell death (Fontana et al., 2018). Moreover, for NPs to function as an efficient delivery system several criteria should be taken into account like those discussed in the following sections.

### Interaction of Nanoparticles With Antigen/Adjuvants

**Figure 2:**
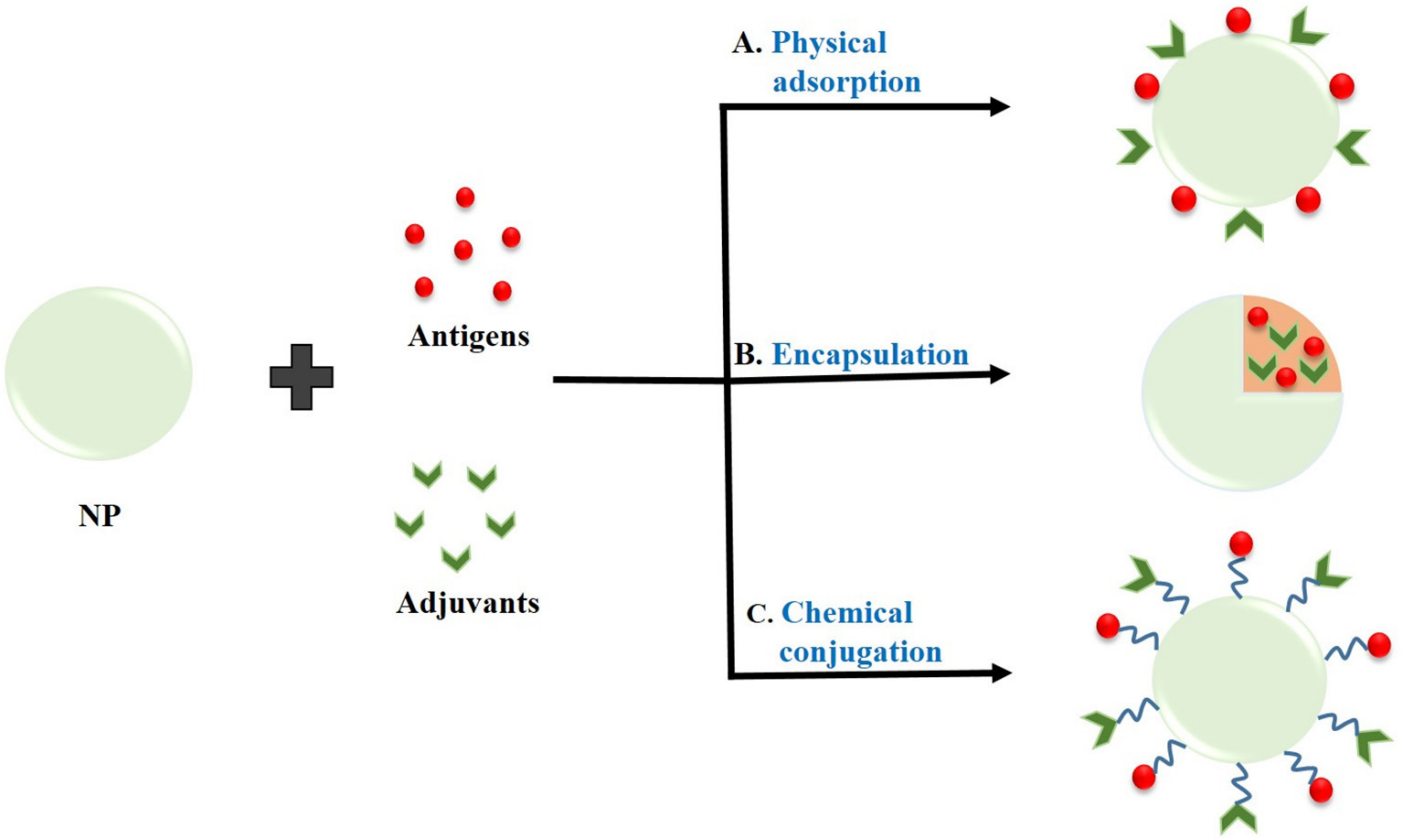
Interaction of nanoparticles (NPs) with antigens or adjuvants by **(A)** Physical adsorption, **(B)** Encapsulation, and **(C)** Chemical conjugation.

The type of interaction between the NPs and antigens for the effective delivery of the antigen is very important in order to protect the cargo molecules from enzymatic degradation and its premature release within the circulatory system. The loading of antigen and/or adjuvant molecules on the NPs has been done through: physical adsorption, encapsulation, or chemical conjugation (Zhao et al., 2014). The different types of interactions between NPs and antigens or adjuvants are shown in Figure 2 as well as illustrated in Table 2.

**Table 2:** Interaction of nanoparticles with antigen/adjuvant.

**S. No**	**Type of interaction**	**Nanoparticle**	**Antigen/adjuvants**	**References**
1.	Adsorption	MSNs	BSA	Hartono et al., 2010
		MSNs	OVA	Mahony et al., 2013
		MWCNTs	OVA and Cancer Testis Antigen, NY-ESO-1	Faria et al., 2014
		MWCNTs	OVA	Dong et al., 2019
2.	Encapsulation	PLGA NPs	Protein antigens and two adjuvants (a TLR2 agonist and a TLR3 agonist)	Rosalia et al., 2015
		Liposomes	OVA	Yuba et al., 2014
		PLGA NPs	OVA and poly (I:C) as an adjuvant	Han et al., 2016
		PLGA NPs	TLR7/8 agonist	Kim et al., 2018
3.	Chemical Conjugation	Trimethyl chitosan	OVA	Slütter et al., 2010
		LDH NPs	Three antigen peptides (Trp2, M27, and M30) and CpG-ODN	Zhang et al., 2018
		GNPs	CpG-ODNs and BSA antigen	Dykman et al., 2018
		Gold nanospheres	CpG-ODNs	Luo J. et al., 2019

#### Physical Adsorption

Adsorption of antigen and/or adjuvant molecules on the surface of NPs is generally carried out through electrostatic interactions or hydrophobic interactions (Wendorf et al., 2006; Mody et al., 2013). This type of interaction is relatively weaker which results in rapid disassociation and release of antigens from NPs inside the cell (Zhao et al., 2014). This type of interaction is directed by suitably engineering the NPs surface with appropriate coating materials or functionalization. Hartono et al. (2010) investigated the adsorption behavior of bovine serum albumin (BSA) on functionalized FDU-12 mesoporous silica surface. They demonstrated that vinyl functionalized FDU-12 silica adsorbed BSA via hydrophobic interactions whereas amino-functionalized FDU-12 silica adsorbed BSA via electrostatic interactions. Later, Mahony et al. (2013) also demonstrated the adsorption of a model antigen, OVA on amino-functionalized MSNs via electrostatic interaction between positively charged amino groups on the surface of NPs and negatively charged protein. In another study, Faria et al. (2014) also reported the non-covalent adsorption of TAA (NY-ESO-1) as well as an adjuvant (CpG-ODN) on the surface of oxidized MWCNTs. Recently, Dong et al. (2019) loaded OVA on the surface of mannose- modified MWCNTs via physical adsorption for its effective and targeted delivery into DCs.

#### Encapsulation

Encapsulation of antigens and/or adjuvants within the polymeric NPs is achieved by simply mixing the antigens and/or adjuvants along with the polymer followed by emulsification and solvent evaporation. On the other hand, encapsulation of antigens and/or adjuvants within the liposomes is achieved by thin film hydration method, dehydration-rehydration techniques or freeze thaw sonication methods. Encapsulation leads to relatively stronger binding of the cargo molecules within the polymeric NPs or liposomes and the trapped molecules are released into the cytosol following internalization into the cell and subsequent NP degradation (He et al., 2000). For example, Yuba et al. (2014) reported the encapsulation of OVA into dextran-modified liposomes which were efficiently taken up by the DCs via endocytosis. After that the antigens were released into cytosol due to rupture of the liposomes in weakly acidic environment of endosomes. Rosalia et al. (2015) formulated a PLGA NP-vaccine by co-encapsulating an antigen (OVA) and two adjuvants Pam3CSK4 (TLR2 ligand) as well as poly (I:C) (TLR3 ligand) coupled with αCD40 antibody to the NP surface for targeted delivery into DCs. In another study, Han et al. (2016) also reported the co-encapsulation of both antigen (OVA) and adjuvant, poly (I:C) within PLGA NPs for efficient delivery into DCs. Later, Kim et al. (2018) encapsulated a novel TLR7/8 agonist within PLGA NPs for their effective delivery into DCs and observed a significant increase in antigen-specific immune response as compared to free TLR7/8 agonist.

#### Chemical Conjugation

Chemical conjugation of antigens and/or adjuvants with NPs provides the strongest interactions, therefore it allows slow release of the cargo molecules. In this type of interaction, the antigen is chemically cross-linked to the surface of the NPs (Zhao et al., 2014). For example, Slütter et al. (2010) covalently linked OVA to N-trimethyl chitosan (TMC) using a reducible disulfide bond to enhance the immunogenicity of the antigens as compared with OVA/TMC NPs. In 2018, Zhang et al. conjugated classical and personalized neoantigens (Trp2, M27 and M30) as well as one immunoadjuvant, CpG-ODN with layered double hydroxide NPs (LDH NPs) and found significant inhibition of melanoma growth (Zhang et al., 2018). They first chemically conjugated Trp2, M27 and M30 peptides with BSA *via* amide bond formation, and then coated the LDH NPs surface by the BSA-antigens. In the same year, Dykman et al. (2018) conjugated GNPs with CpG-ODNs via coordination bond between GNPs and 5′-thiolated ODNs, and investigated their immune response toward antibody production. In another study, thiol- modified CpG-ODNs were conjugated on the surface of hollow gold nanospheres via Au-S bonding for their effective delivery and enhancing the immunostimulatory effect of CpG-ODNs (Luo J. et al., 2019).

### Factors Influencing the Interaction of Nanocarriers With Antigen-Presenting Cells (APCs)

**Figure 3:**
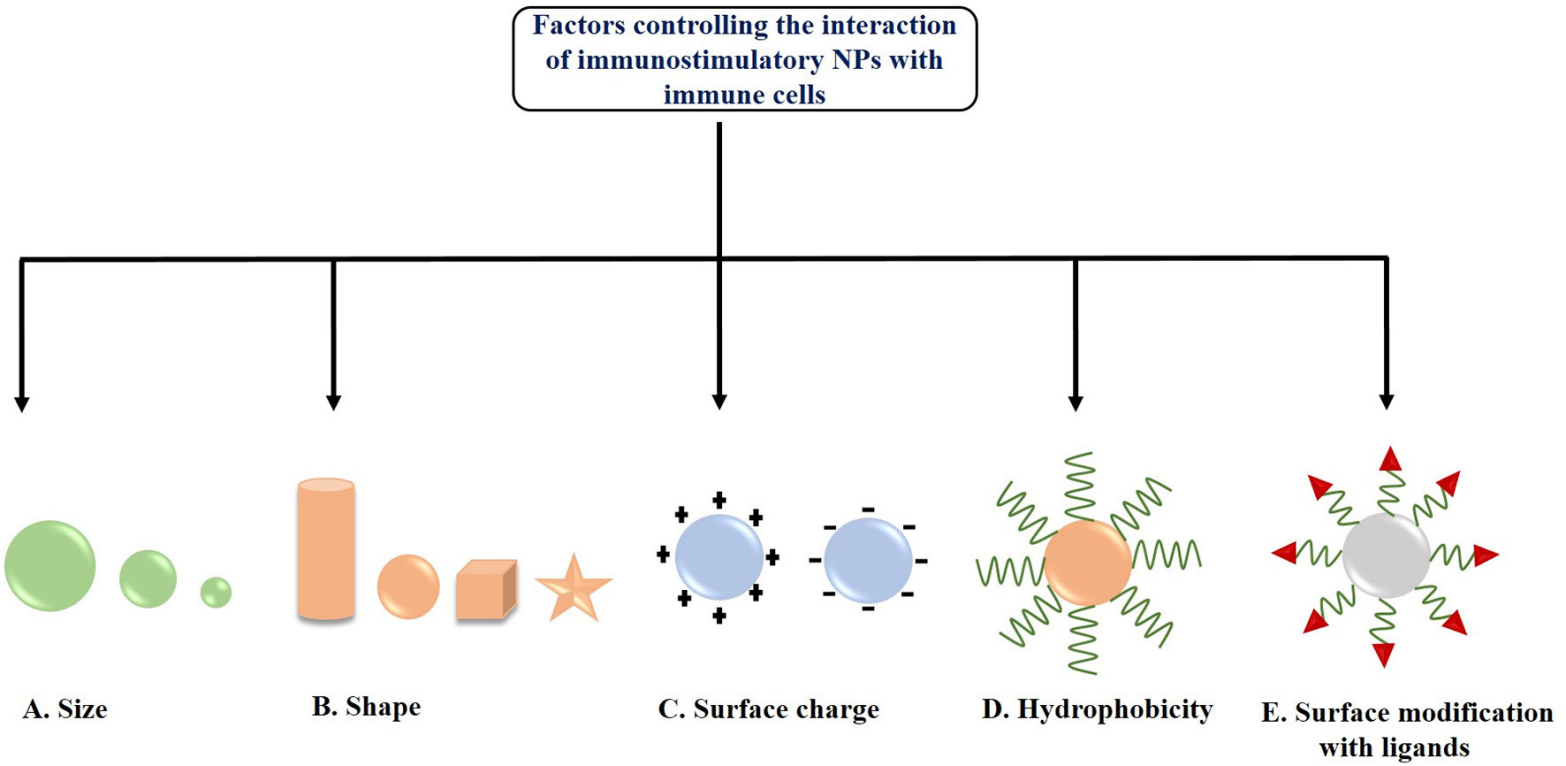
Factors controlling the interaction of immunostimulatory nanoparticles (NPs) with immune cells **(A)** Size, **(B)** Shape, **(C)** Surface charge, **(D)** Hydrophobicity, and **(E)** Surface modification with ligands.

There are certain cells among the immune system in our body, which help the killer cells, like Natural Killer cells (NK cells), or cytotoxic T-cells, to recognize the presence of foreign or malignant-origin antigens in our body. They process them upon specific recognition, and rather than destroying it, they present them to the killer cells for an enhanced and exponential immune reaction. These are the Antigen-presenting Cells or APCs, and include B-cells, macrophages, DCs and Langerhans cells. The APCs mediate immune response in various ways, such as by producing immunoregulatory cytokines, or antibodies (in case of B-cells), as well as activating CD4^+^ helper T-cells, which modulate immunological activity by releasing cytokines, or by activating CD8^+^ cytotoxic T cells by themselves acting as APCs. Here it is to be noted that CD4^+^ helper T-cells are only activated upon recognition of a non-self or malignant antigenic peptide, presented upon MHC-II molecules displayed on the cell surface exclusively by the APCs. In order to induce anti-tumor immune response, the tumor antigens and adjuvants must be effectively delivered to APCs *via* a favorable interaction between the nanocarriers and the APCs. This interaction is highly dependent upon several factors such as NPs size, shape, hydrophobicity, surface functionalization and surface charge (Zhao et al., 2014; Park et al., 2018). The factors controlling the interaction of immunostimulatory NPs with immune cells are depicted in Figure 3.

#### Size

The size of NPs is one of the major factors influencing its interaction with the APCs or DCs located in the lymph nodes and its subsequent internalization. Park et al. (2018) reported that medium-sized NPs (~5–100 nm) were more effectively delivered to lymph nodes as compared to small (<5 nm) and large sized (>100 nm) NPs. In another study, Reddy et al. (2006) demonstrated that smaller NPs (20 nm) were more readily taken up into the lymphatic network and reached the lymph node as compared to larger NPs having size 45 and 100 nm. Hirai et al. (2012) checked the effects of NPs size on cross-presentation in DCs. They demonstrated that silica NPs having diameters 70 and 100 nm were able to enhance the entry of exogenous antigens in the cytosol and thus induced cross-presentation, whereas large NPs having diameter >100 nm did not. Besides, García et al. (2013) also studied the size-dependent interaction of GNPs with THP-1 human monocyte cells. They showed that the internalization of larger GNPs (15 and 35 nm) was blocked in presence of latrunculin-A (a phagocytosis inhibitor), whereas smaller NPs (5 nm) were evenly uptaken and not blocked by actin-dependent processes. Moreover, Kang et al. (2017) synthesized three different-sized OVA loaded GNPs (OVA- GNPs) having hydrodynamic diameters 10, 22, and 33 nm and found a size-dependent cellular uptake by DCs. 22 and 33 nm OVA-GNPs showed much higher delivery efficiency in lymph nodes upon injection into mouse footpad and exhibited higher induction of CD8^+^ T cell responses as compared to 10 nm OVA-GNPs as observed in *ex vivo* restimulation assay. Further, the tumor-prevention study demonstrated that 22 nm OVA-GNPs showed higher antitumor efficacy and greater tumor cell apoptosis as compared to 10 nm OVA-GNPs. All these findings suggest that the effective delivery of antigens *via* nanocarriers is controlled *via* a size-dependent interaction of NPs with APCs.

#### Shape

Along with size, the shape of the NPs is another key factor for effective interaction with the cell membrane of APCs as well as their internalization (Champion and Mitragotri, 2006; Toy et al., 2014). In 2013, Niikura et al. (2013) studied the effect of West Nile virus (WNV) envelope (E) protein coated GNPs with different shapes like spherical, cubic, and rod-like on immunological response in RAW264.7 macrophages and BMDCs. The results demonstrated that rod-shaped GNPs were more efficiently uptaken by the cells as compared to spherical and cubic GNPs. Further, it has also been observed that rod-shaped NPs induced the production of IL-1β and IL-18 (inflammasome – related cytokines), whereas cubic and spherical shaped NPs induced the production of IL-6, IL-12, TNF-α, and granulocyte-macrophage colony-stimulating factor (pro-inflammatory cytokines) from BMDCs. Another immunization study by Kumar et al. (2015) showed the effective interaction of both spherical (193 nm in diameter) and rod-shaped antigen-carrying polystyrene NPs (1,530 nm in length) with DCs. It has been observed that both NPs were internalized into DCs and effectively delivered OVA. Moreover, this study also demonstrated that spherical polystyrene particles generated Th1-biased immune response whereas rod-shaped particles generated Th2- biased immune response against OVA. Besides, Dykman et al. (2018) synthesized GNPs of different size and shapes such as, nanospheres (15 and 50 nm diameter), nanoshells, nanostars as well as nanorods and conjugated them with a model antigen, BSA and adjuvant, CpG-ODNs for studying their effects on the production of antibodies. They found gold nanospheres (15 and 50 nm diameter) conjugated with CpG-ODNs as the optimal antigen carrier as well as adjuvant for immunization. All these studies are clearly indicating the shape-dependent immunological responses of the NPs.

#### Surface Charge

In addition to the size and shape of NPs, the surface charge of the nanocarrier also plays a significant role in effective interaction with APCs. Generally, positively charged NPs have been found to induce higher DC uptake due to electrostatic interaction with negatively charged cell membranes generating a higher immune response (Foged et al., 2005). Nakanishi et al. (1999) demonstrated that positively charged liposomes effectively delivered chicken egg albumin into the cytoplasm of APCs and induced higher degree of antigen-mediated immune responses due to increased interaction and consequent higher uptake as compared with negatively charged and neutral liposomes. Furthermore, Yan et al. (2008) reported that in contrast to neutral liposomes, cationic liposomes produce ROS inside DCs and this ROS is further required for ERK and p38 activation as well as induction of downstream cytokines, chemokines, and co-stimulatory molecules. Recently, Srijampa et al. (2020) demonstrated that positively charged GNPs exhibited a high potential to induce immune responses (induce both pro-inflammatory, IL-1β and anti-inflammatory, TGF-β cytokine expression) as compared to negatively charged GNPs (induce only pro-inflammatory, TNF-α cytokine expression) in human monocyte cells. Although cationic NPs are suitable for cellular internalization through electrostatic interaction but sometime cationic NPs can cause platelet aggregation and hemolysis or can disturb membrane integrity. Therefore, by coating the NPs surface with hydrophilic molecules such as PEG, the excess positive charge can be balanced (Grimaldi et al., 2017). Various studies also proved that PEGyaltion can be a beneficial strategy to enhance the NPs interactions with DCs (Fahmy et al., 2008; Zhuang et al., 2012), and it also induces the complement activation (Szebeni et al., 2002; Moein Moghimi et al., 2006). In addition to PEG, polyethylene oxide can also be used as a coating agent for NPs, but it is not suitable because of its toxic nature (Getts et al., 2015).

#### Hydrophobicity

Several researchers also demonstrated that hydrophobicity of the nanocarriers is an important factor in inducing immune response. Raghuvanshi et al. (2002) reported that hydrophobic polymeric NPs induced higher immune response as compared to hydrophilic polymeric NPs during immunization with tetanus toxoid. In another study, Moyano et al. (2012) checked the direct relation between hydrophobicity of NPs and their immune responses. They functionalized GNPs with different degree of hydrophobicity to examine their effect on the immune response in splenocytes (*in vitro*) and in mice (*in vivo*) by checking the cytokine expressions, and observed a linear increase in the immune activity with increasing hydrophobicity. Shima et al. (2013) also studied the effect of hydrophobicity of amphiphilic poly(g-glutamic acid)-graft-L-phenylalanine ethyl ester (γ-PGA-Phe) NPs on the induction of immune responses in DCs. They observed that an increase in hydrophobicity of these NPs enhanced the antigen-bound NP uptake by DCs, activation of DCs, and induction of antigen-specific immune response. In another study, Shima et al. (2015) synthesized OVA encapsulated-NPs from amphiphilic PGA with various types of hydrophobic segments. They observed that the NPs significantly increased the interactions with DCs as well as induced antigen-specific immune response which strongly depends upon the type of hydrophobic segments and the vaccine formulations used. Hence, all these studies suggest a direct correlation between hydrophobicity of nanocarriers and immune system activation.

#### Surface Modification With Ligands

Modification of the surface of NPs with specific ligands is another important factor that enhances the targeted delivery of NPs to APCs or DCs, thereby preventing its off-target side effects and inducing highly effective anti-tumor immune response (Jo et al., 2017). For example, a study conducted by Shi et al. (2017) demonstrated that mannose-decorated chitosan NPs could serve as antigen delivery vehicles and specifically target DCs under both *in vitro* and *in vivo* conditions. The NPs-loaded antigens further triggers DC maturation, anti-tumor cytokine release as well as tumor growth inhibition. In another study, amino-functionalized MSN was covalently coupled with peptide TY and further loaded with OVA/CpG-ODNs for targeted co-delivery of antigen and adjuvant to DCs. Their results showed that such DC targeting nanosystem enhanced antigen uptake as well as promoted the activation and maturation of DCs which further triggered T-cell mediated immune response to eliminate tumor with less systemic toxicity (Liu et al., 2019).

## Strategies for Immunostimulatory Nanoparticles in Cancer Immunotherapy

Cancer immunotherapy faces the major challenge of delivering antigens for the subsequent induction of immune response (Zang et al., 2017). Sufficient antigen/adjuvant is required to trigger naïve T-cell differentiation/activation and antigen presentation by the APCs for the subsequent activation of CD8^+^ and CD4^+^ T cells (Palucka and Banchereau, 2013; Melero et al., 2014; Zhang et al., 2020). APCs are immune cells which present the antigens to the epitopes of class I and II major histocompatibility complex (MHC) molecules displayed on the killer cells' surface, to interact with T cell receptors. Our immune system has four major types of APCs (1) DCs, (2) B cells, (3) macrophages, and (4) monocytes. Besides these, there are some amateur APCs that function under certain conditions, such as vascular endothelial cells, thymic epithelial cells, fibroblast, pancreatic β cells, and glial cells (Otsuka et al., 2020).

These days, researchers have paid more attention to engineer immunostimulatory NPs which can be effectively internalized by the APCs, thereby enhancing the immunotherapeutic efficacy via stimuli-responsive and selective delivery of cancer antigens/adjuvants into the target cells to initiate the antigen-specific immune response, and by reprogramming TME (Zhang et al., 2017; Chiang et al., 2018; Feng et al., 2019). These NPs allow serum proteins to bind to its surface and form a corona to interact with various receptors and produce desired immunostimulatory effects (Liu et al., 2018). Besides targeted delivery, NPs could also protect the cargo molecules from bioactivity loss during circulation, avoid off-target side effects (Feng et al., 2019).

### Targeting/Stimulation of Dendritic Cells (DCs)

DCs are the major APCs that play an important role in innate and adaptive immunity (Manh and Dalod, 2016). Upon activation through antigens/pathogens, they further stimulate T-cell activation and B-cell differentiation (Eisenbarth, 2019). The origin of DCs is bone marrow and it is further classified into plasmacytoid DCs (pDCs) and myeloid DCs (mDCs). DCs express both MHC-I and MHC-II molecules and present the antigens to the T-cell receptors to activate CD8^+^ and CD4^+^ T cells respectively (Sabado et al., 2017).

**Figure 4:**
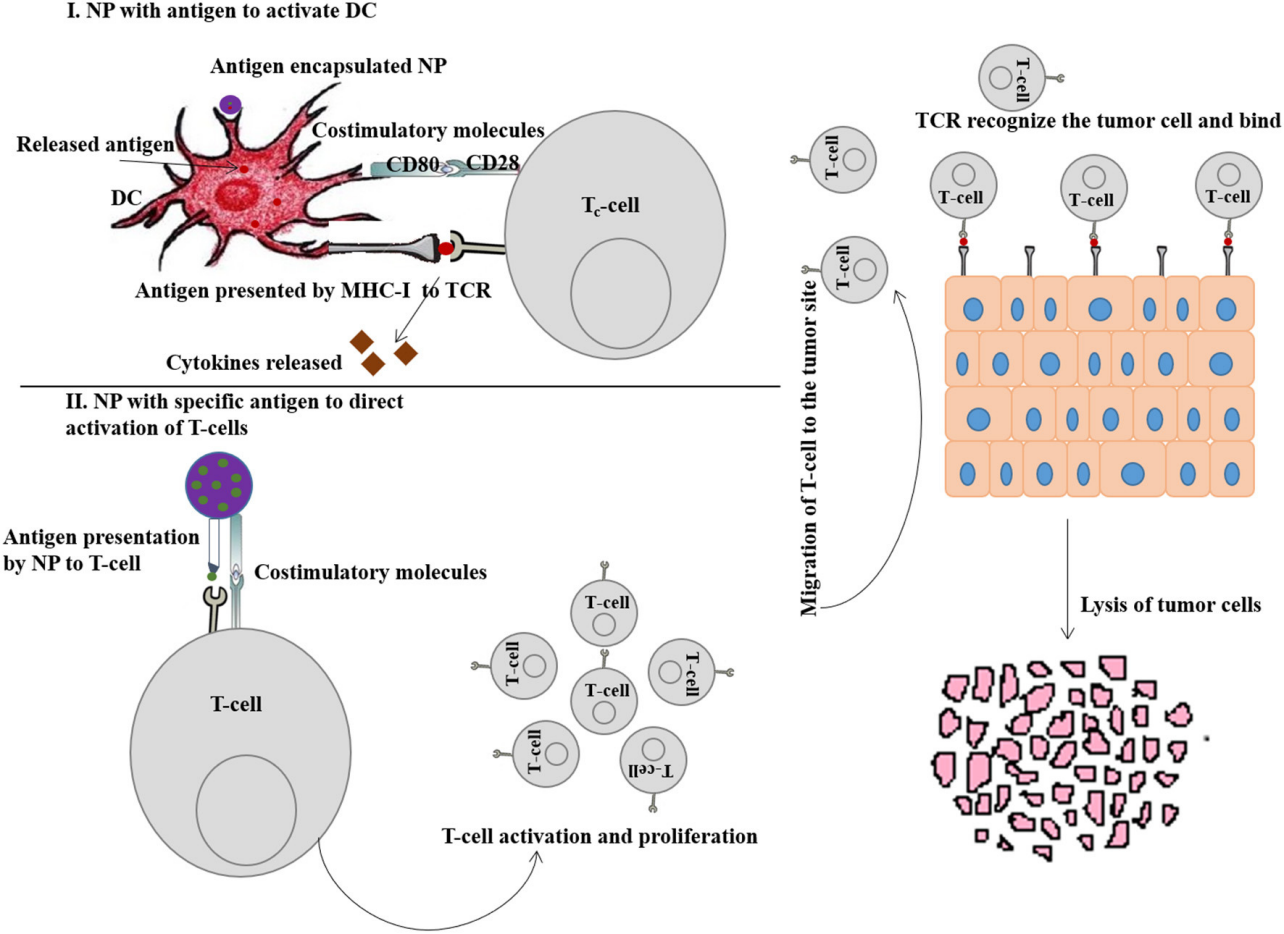
(I) Schematic representation of DC activation by immunostimulatory nanoparticle (NP) to enhance cancer immunotherapy. (II) Schematic representation of T-cell activation by immunostimulatory NP to enhance cancer immunotherapy.

DCs possess TLRs which play an important role in both innate and adaptive immune response by the recognition of intracellular and extracellular pathogens. Activation of TLR signal transduction pathways facilitates adaptive immune response by the production of cytokines, chemokines and MHC molecules (Fitzgerald and Kagan, 2020). Delivery of appropriate TAAs to DCs via NPs is imperative to instigate T-cell responses for subsequent cancer immunotherapy. Figure 4 (I) represents the DC activation pathway by immunostimulatory NP for enhanced cancer immunotherapy.

**Figure 5:**
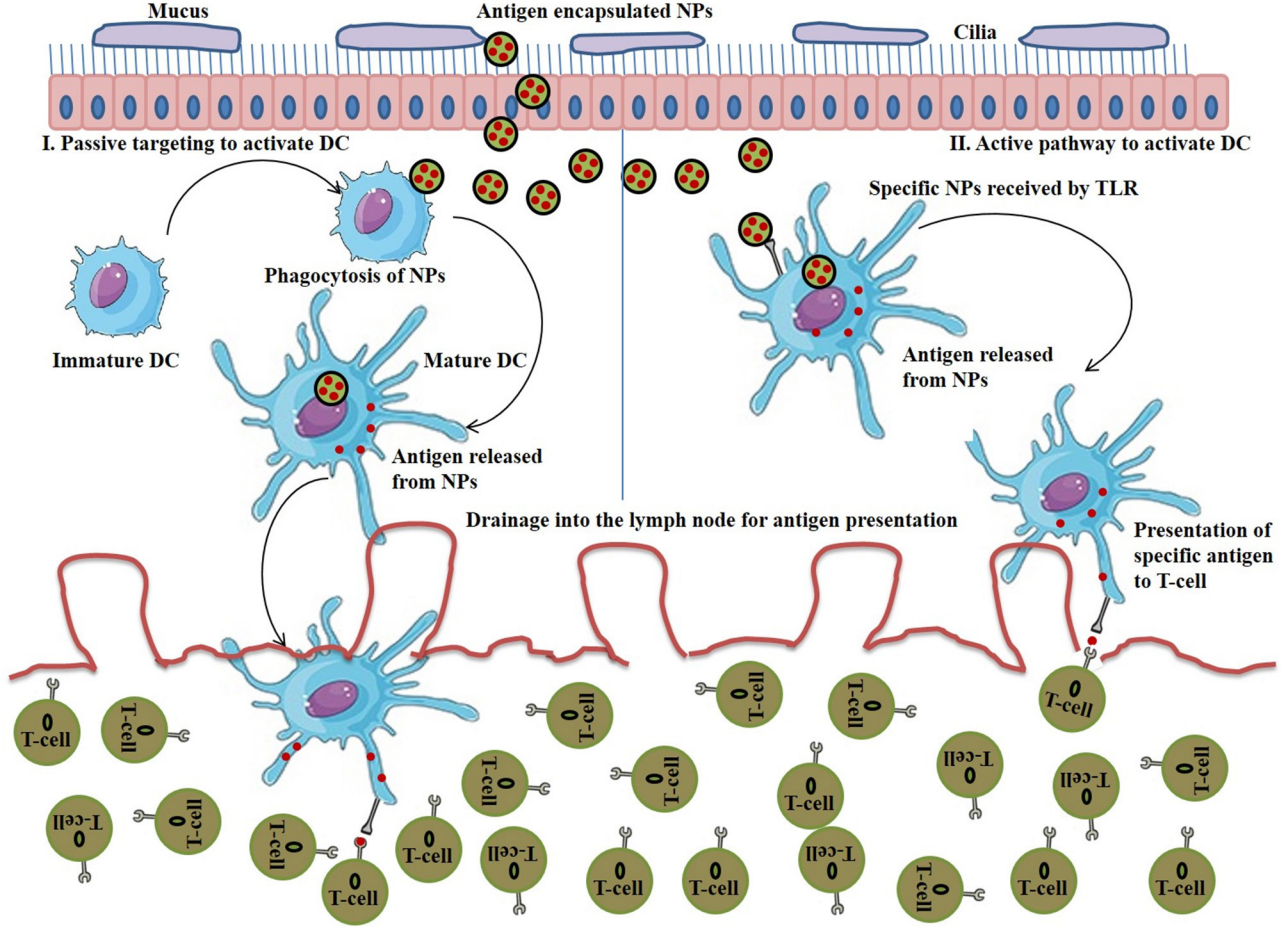
(I) Stimulation of passive pathway to activate DC for cancer immunotherapy by immunostimulatory nanoparticles (NPs), (II) Stimulation of active pathway to activate DC for cancer immunotherapy by immunostimulatory NPs.

The majority of DCs are found in lymphoid organs (spleen and lymph nodes) and also occupied by peripheral tissues (Bryant et al., 2019). NPs bound with antigens/adjuvants are internalized through endocytosis, and if the antigens are not released from the endosomes into cytosol, they present the antigens to the MHC-II pathway and activate the CD4^+^ T cells (Tran et al., 2018). Therefore, for effective immunotherapy, antigen should be bound with MHC-I molecules which requires the release of antigen into cytosol and formation of antigen-MHC-I complex within endoplasmic reticulum to activate CD8^+^ T cells (Colbert et al., 2020). Stimulation of DCs can be achieved either through passive targeting by directing antigen-loaded NPs toward sites that are rich in DCs or by active targeting. Nanovaccines are typically delivered via subcutaneous routes and easily drain (NPs with size <100 nm) into the lymph nodes for antigen presentation. However, large sized NPs (size > 500 nm) are mostly trapped at the site of administration and can only be taken up by skin-resident DCs and transport them to lymph nodes. NPs within the size ranging from 100 to 500 nm show both free and cell-based drainage to lymph nodes. Therefore, NP size is considered to be an important factor when designing nanovaccines for passive transport to DCs and subsequent uptake. The other important factors are surface charge, hydrophobicity and morphology of NPs (Tran et al., 2018), discussed in the earlier sections. Figure 5 (I) represents the passive pathway to activate DC for cancer immunotherapy by immunostimulatory NPs.

On the other hand, to initiate the stimulation of DCs via active targeting, nanovaccines are functionalized with specific antibody or ligands that explicitly bind to DC's surface receptors such as C type lectin receptors, mannose receptor, DCIR2, CLEC9A, DEC205, and Langerin (Kreutz et al., 2013). Surface TLRs (TLR 1, 2, 4, 5, 6, and 10) are another promising candidate to target APCs. On the other hands, the ligands specific for intracellular TLRs (TLR 3, 7, 8, and 9) are used as vaccine adjuvants for the stimulation of DCs and T-cells activation in nanovaccine-mediated immunotherapy (Tran et al., 2018). Figure 5 (II) represents the active pathway to activate DC for cancer immunotherapy by immunostimulatory NPs.

### Targeting/Stimulation of T-Cells

Direct stimulation or activation of T-cell *via* NPs is an alternative immunotherapeutic approach instead of stimulation via DCs. Typically, there are two strategies to stimulate T-cells activation: (1) expansion of T-cells isolated from lymphocytes of a cancer patient under *ex vivo* condition and then reinsertion of these T-cells into the patient (Broere and van Eden, 2019), and (2) to design nanovaccines for the tumor-specific CD8^+^ T cells activation (Zhang et al., 2017). In both the cases, artificial APCs (aAPCs) are required to mimic the antigen-presentation as well as T-cell activation capacity of natural APCs. However, in comparison to the cell-based aAPCs, acellular or NP-based aAPCs possess significant advantages in avoiding the development of immune response against cell-based aAPCs. Therefore, here we would like to discuss the recent progress of NP-based aAPCs in direct activation of T-cells.

Several nanoformulations including liposomes, magnetic beads, paramagnetic NPs and biodegradable polymers have been engineered to synthesize aAPCs for direct T-cell activation (Zang et al., 2017). The essential signals required for T-cell activation by aAPCs are (1) co-stimulatory signal, like anti-CD3 or anti-CD28 antibodies, and (2) peptide-MHC-I molecules (Kosmides et al., 2017). Apart from that, conjugation with pro-inflammatory cytokines and immune-checkpoint antibodies can further stimulate and enhance the anti-tumor effects of T-cell. Figure 4 (II) represents the T-cell activation pathway by immunostimulatory NP for enhanced cancer immunotherapy.

Zhang et al. (2017) reported the use of magnetic nanocluster coated with azide-modified leukocyte membrane fragments, and functionalized with MHC-I peptide and anti-CD28 antibodies to stimulate T-cell response for cancer immunotherapy.

Immune-checkpoint molecules, such as programmed death-1/programmed death ligand-1 (PD-1/PD-L1) and CTLA-4 play a major role in suppressing the T-cell activation (Hu et al., 2017). Therefore, immune-checkpoint antibodies, such as anti-CTLA-4 or anti-PD1 antibodies are used to block the immune-checkpoint pathways by directly conjugating with CTLA-4 or PD-1 molecules, leading to enhanced T-cell activation (Mahoney et al., 2015). Li S. et al. (2018) reported that conjugation of CTLA-4 antibody with poly (lactic-co-hydroxymethyl-glycolic acid) (PLHMGA) and PLGA exhibited higher anticancer immunotherapy by blocking the inhibitory receptor on T-cells. Kosmides et al. (2017) used PLGA–derived aAPCs conjugated with anti-PD1 monoclonal antibody for synergistic effects on CD8^+^ T cell activation and tumor growth inhibition. Ye et al. (2016) developed microneedle-based transcutaneous delivery of self-assembled amphiphilic hyaluronic acid NPs loaded with indoleamine 2,3- dioxygenase (IDO) inhibitor, 1-methyl-DL-tryptophan (1MT), and anti-PD-1 antibody for enhanced-cell immunity and decreased immune-suppression.

Mi et al. (2018) synthesized a dual immunotherapy nanoparticle (DINP) using PEG-PLGA conjugated with both anti-OX40 (T cells agonist antitumor necrosis factor receptor superfamily member 4) and anti-PD-1 antibodies. This DINP induced higher T-cell activation and showed effective anti-tumor activity in B16F10 melanoma tumor model as compared with monotherapy. Further, a polysaccharide-based SPION conjugated with anti-CD3, anti-CD28, and anti-PD-L1 antibodies has been developed by Chiang et al. (2018) for the synergistic antitumor effect via immune-checkpoint inhibition and T-cell activation.

Several cytokines, such as IL-2 also stimulate T-cell activation. IL-2 helps in cluster initiation of T-cell and its persistence after antigen priming (Abbas et al., 2018). Fadel et al. (2014) used a CNT-polymer composite functionalized with MHC-I peptide, anti-CD28, and IL-2 for enhanced T-cell expansion and inhibition of tumor growth in mice with melanoma.

Park et al. (2012) showed that IL-2 encapsulated liposomal polymeric gels along with TGF-β inhibitor enhanced the CD8^+^ T cell infiltration and inhibited the tumor growth (Park et al., 2012). Along with IL-2, IL-12 can also activate T-cells. Several researchers have shown that IL-12 loaded on polysaccharide chitosan (Zaharoff et al., 2010; Smith et al., 2015), pH-responsive poly (β-amino ester) copolymers (Wang et al., 2017), cholesterol-bearing pullulan (CHP)- based hydrogel NPs (Shimizu et al., 2008), biodegradable poly(lactic acid) microsphere (Kilinc et al., 2006), and PLGA matrices (Ali et al., 2014) promoted T-cell dependent tumor regression.

Plasmids encoding immunostimulatory cytokines can also be delivered *via* NPs for enhanced cancer immunotherapy. Small interfering RNA (siRNA) also act as T-cell stimulators by downregulating the expression of immunosuppressor molecules. For example, PEG-PLA NPs [poly(ethylene glycol)-block-poly(D,L-lactide)] loaded with CTLA-4 siRNA efficiently restored the T-cell functions by downregulating CTLA-4 level and increasing the CD4^+^ and CD8^+^ T cell populations as well as inhibited tumor growth in mice with melanoma (Li S.-Y. et al., 2016).

### Targeting/Stimulation of Natural Killer Cells

Natural killer cells (NK cells) are large granular lymphocytes and play a key role toward both innate and adaptive immunity. They contribute 5–15% of circulating lymphocytes and have various subpopulations depending upon the maturation site. They play a protective role against cancer and infectious-pathogens like viruses, and get activated in the absence of the inhibitory signal. The inhibitory receptors expressed by NK cells such as, killer immunoglobulin-like receptors (KIR2DL1/2/3 in humans), Ly49A/C (in mice), and CD94-NKG2A heterodimer (in both humans and mice) bind with MHC class I molecules of normal healthy cells and contributes to the self-tolerance for host cells. But in malignant or virus-infected cells, the expression of these MHC class I molecule is downregulated, leading to lower inhibitory signals in NK cells. In addition, the cellular stress associated with tumor development or infection also causes upregulation of ligands for activating receptors such as NKp30, NKp44, NKp46, NKG2D, LY9D, etc. on NK cells for their activation. Upon activation, NK cells eliminate the target cells either directly *via* releasing cytotoxic granules with granzyme B and Perforin or indirectly *via* the release of pro-inflammatory cytokines. NK cells also mediate the antibody-dependent cellular cytotoxicity (ADCC) when the low-affinity activating receptor FcγRIIIa (CD16) of NK cells binds with the Fc portion of immunoglobulin G1 (IgG1) (Fang et al., 2017, Guillerey et al., 2016). Beside its killing effect, NK cells also regulate the immune response against tumor cells. It secretes some chemokines and pro-inflammatory cytokines that can regulate the DCs and T-cells response (Fang et al., 2017, Guillerey et al., 2016).

NK cell-based anticancer immunotherapy is highly promising, but faces insurmountable challenges such as short *in vivo* life span and poor expansion of NK cells, as well as cost and complexities in treatment methods. However, particle-based NK cell expansion strategies have efficiently overcome these limitations by delivering various NK cell-stimulating molecules that can activate the NK cells; for example, plasma membrane particles derived from K562-mbIL15-41BBL and K562-mb21-41BBL cells expressing 41BBL as well as membrane bound interleukin-15 (PM15 particles) and interleukin-21 (PM21 particles) selectively expand NK cells that exhibit significant cytotoxicity against the leukemia cells (Oyer et al., 2015, 2016); nanoscale graphene oxide linked with anti-CD16 antibody targets the CD16 receptor of NK cells, thereby leading to their activation (Loftus et al., 2018). Park et al. (2017) reported the use of magnetic microspheres with PLGA and recombinant IFN-γ for imaging-guided cancer immunotherapy. The intra-arterial transcatheter delivery of magnetic microspheres into liver tumors significantly enhanced the NK infiltration into tumors via IFN-γ dependent chemokine (CXCL10) secretion. In another study, Gao et al. (2020) developed tumor-targeting RGD peptide-modified diselenide-containing polymeric NPs for their effective accumulation into tumor via systemic administration and NK cell-mediated cancer immunotherapy via radiation exposure. Upon radiation exposure, diselenide portion in the polymer is oxidized to seleninic acid, which can enhance the NK cell-mediated immunomodulatory activity by blocking the interaction between human leukocyte antigen-E (HLA-E) and inhibitory checkpoint receptor, NKG2A.

NK cells can also be co-delivered with NPs to inhibit the spread of tumor cells or metastasis. Chandrasekaran et al. (2016) showed that TRAIL-decorated liposomes conjugated to NK cells within the tumor draining lymph nodes (TDLN) prevented the metastasis of a subcutaneous tumor in mice. They also reported that TRAIL functionalization on NK cells increased their retention time within TDLN to induce apoptosis of tumor cells. Later, Wu et al. (2018) have demonstrated the magnetic targeted delivery of NK cells loaded with Fe_3_O_4_@polydopamine NPs into tumor for improved cancer immunotherapy (Wu et al., 2018).

Activation of TLRs in DCs results in increased co-stimulatory molecule upregulation and pro-inflammatory cytokine secretion, both of which can help to activate NK cells. Kim H. et al. (2020) have developed polymeric NPs, encapsulating imidazoquinoline-based TLR7/8 agonist which promotes prolonged NK cell activation *in vivo*. Delivery of the TLR7/8 agonist-loaded NPs results in enhanced ADCC with cetuximab antibody and increases its antitumor efficacy in mice tumor model (Kim H. et al., 2020).

Delivery of genes encoding the activating receptors on NK cells and proinflammatory cytokines *via* NPs can stimulate NK cell activation for improved cancer immunotherapy. Tan et al. (2017) reported the synthesis of chitosan-based NPs for the delivery of fused dsNKG2D–IL-21 gene encoding both NKG2D and IL-21 genes. Treatment with the dsNKG2D–IL-21 gene NPs caused increased secretion of serum IL-21 and activation of NK cells which retarded the growth of tumor and increased the life span of tumor-bearing mice. In another study, Meraz et al. (2018) delivered plasmid DNA encoding tumor suppressor candidate 2 (TUSC2) gene using cationic liposomes in syngeneic Kras-mutant mouse lung cancer models. Treatment with TUSC2 significantly inhibited tumor growth and increased the survival of mice *via* the upregulation and activation of the NK and CD8^+^ T cells in the blood and TME. However, combination treatment with anti-PD1 antibody synergistically enhanced its anticancer efficacy.

### Regulating Immune-Suppressive Tumor Microenvironment (TME)

The TME consists of various types of cells apart from malignant cells such as TAMs, myeloid-derived suppressor cells (MDSCs), lymphocytes, pericytes, fibroblasts, the tumor vasculature endothelial cells, and sometimes adipocytes (Roma-Rodrigues et al., 2019). These cells interact with some secreted proteins like galectin-3, matrix metalloproteinases (MMP), osteopontin and, TGF-β responsible for cancer development and produce an extracellular matrix (ECM) that creates an environment to spread cancer (Jia et al., 2017). The cancer immunotherapy is adversely influenced by the immunosuppressive nature of TME because of the presence of TAMs, regulatory T cells (Tregs), and MDSCs, together with enzymes and cytokines (TGF-β, indoleamine 2,3-dioxygenase (IDO), IL-10, etc.) (Quail and Joyce, 2013; Musetti and Huang, 2018). Besides, TME also promotes the proliferation and metastasis of cancer cells. However, the anticancer immunity can be enhanced by regulating TME through various strategies (Roma-Rodrigues et al., 2019).

NPs play a major role in regulating the TME. A number of evidences suggest that NPs with tumor therapeutics offer a suitable means to suppress the TME and can further improve the efficacy of cancer immunotherapy. Besides, NPs are also able to transport immunotherapeutic agents to the deeper tumor site to attain improved efficacy (Musetti and Huang, 2018). NPs should be designed in such a way so that they can respond to the biochemical differences that exist between tumor and adjacent tissues, thereby selectively deliver the immune stimulants to the targeted cells within TME. The different strategies include (1) producing hypoxia-responsive NPs; (2) inserting pH-sensitive materials into NPs; (3) use of the enhanced permeability and retention (EPR) effect by controlling NP size and; (4) inserting substrates for intratumoral MMPs (Dewitte et al., 2014; Uthaman et al., 2018). The hypoxic TME is characterized by decreased oxygen pressure (5-10 mmHg). Thus, by inserting oxygen-sensitive elements such as azobenzene or 2-nitroimidazoles into NPs, hypoxia can be utilized for deshielding of PEGylated NPs, enhancing cellular uptake and drug release (Perche et al., 2014; Thambi et al., 2014). On the other hand, MMPs are also considered as an interesting trigger for size-changing NPs. For example, Wong et al. (2011) used 100 nm gelatin NPs for its effective delivery at the tumor site via EPR effect. After extravasation, MMP-2 and MMP-9 degraded the 100 nm gelatin core NP into smaller 10 nm particles which penetrated deep into tumor tissue *via* migration through the tumor's ECM (Wong et al., 2011).

**Figure 6:**
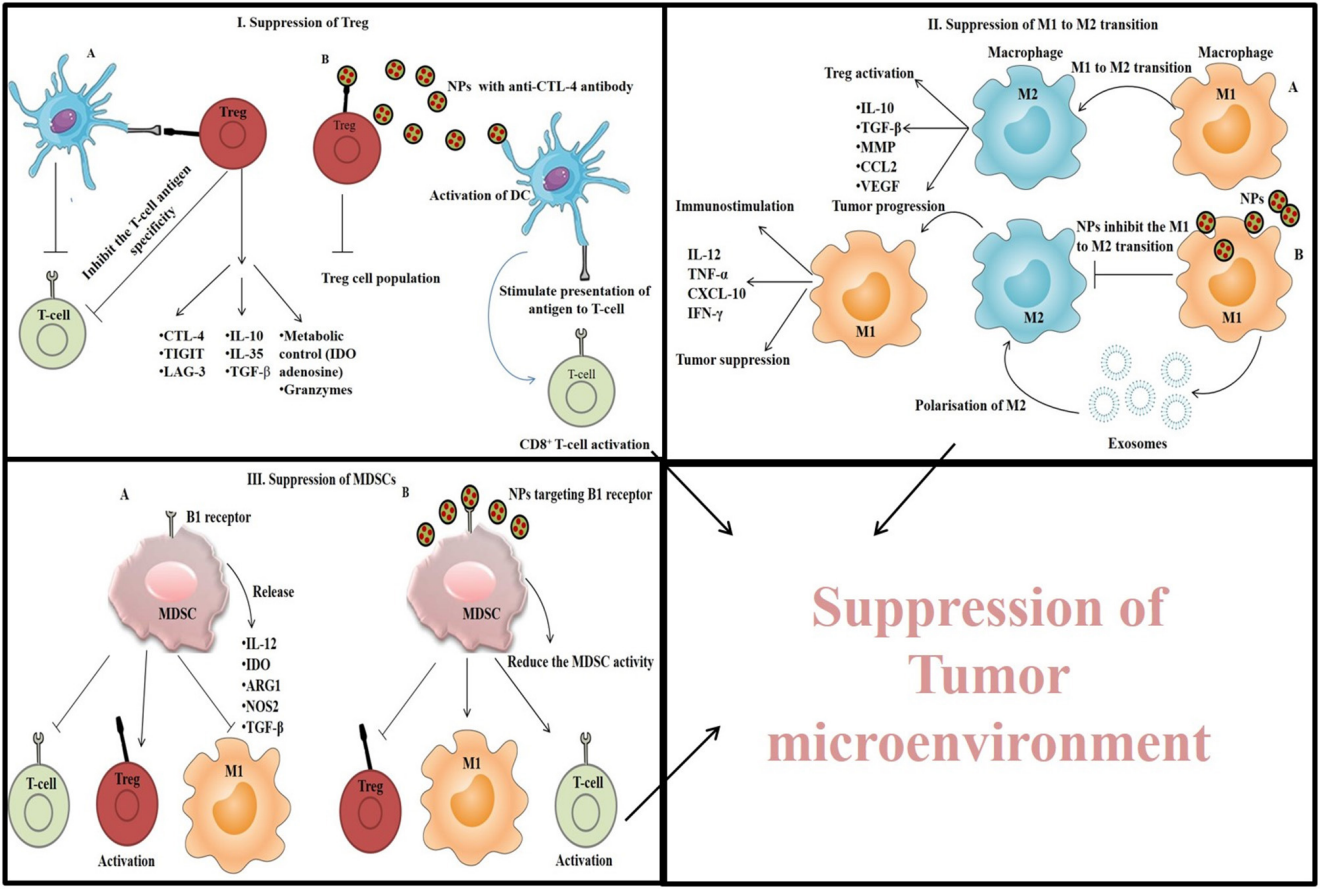
Immunostimulatory NPs mediated TME suppression by (I) targeting Treg and suppressing its function, (II) by inhibiting M1 to M2 transition of macrophages, and (III) by suppressing the MDSC cells.

In TME, T-regulatory cells or Tregs, suppress the immune response by arresting APC function, and inhibiting T-cell activation and proliferation (Jonuleit et al., 2016). Thus, by suppressing the function of Tregs, anti-tumor immunity can be restored (Ou et al., 2018). Immunostimulatory NPs mediated TME suppression by targeting Treg is depicted in Figure 6 (I). A very common strategy to control Treg function is the use of checkpoint blockade antibodies (anti-CTLA-4). Ou et al. (2018) synthesized core-shell NPs (PLGA as core and lipid as shell) conjugated with peptide tLyp1 for targeted delivery of imatinib combined with anti-CTLA-4 antibody. These NPs decreased the intratumoral Treg cell population, increased the CD8^+^ T cell population, and showed a strong antitumor effect against B16 cells xenograft tumors (Ou et al., 2018). The high expression of CD25 on Tregs in TME is another target to enhance the CD8^+^ T cell activation *via* using anti-CD25 antibodies for suppressing the tumor progression (Sato et al., 2016).

TME altered the normal functioning of infiltrated DCs, thereby stimulating Tregs to produce immunosuppressive cytokines and induce apoptosis of CD8^+^ T cells *via* PD-L1. Therefore, intratumoral DCs can be targeted *via* NPs to re-establish their antigen-presenting capacity (Motz and Coukos, 2013). For example, Cubillos-Ruiz et al. (2009) used PEI complexed with PD-L1 siRNA to suppress PD-L1 expression and activation of CD8^+^ T cells by regulating the function of DCs. Besides, PEI also stimulated TLR5 and TLR7 to activate the cells. Similarly, Dominguez and Lustgarten (2010) used poly-lactic acid NPs conjugated with anti-CD40 antibody which target CD40 expressed by DCs, B-cells and macrophages, leading to upregulation of pro-inflammatory cytokines as well as reduction in Treg cell population.

Another target to suppress the TME is TAMs, a population of macrophages that promote tumor growth by protecting cancer cells from chemotherapeutics, attracting Tregs and, inducing apoptosis in CD8^+^ T cells. TAMs are of two types and show either immunostimulation by M1 type of macrophages via the secretion of pro-inflammatory cytokines like TNF-α, IL-12, IL-1b, IL-6, and IFN-γ or immunosuppression by M2 type of macrophages via the secretion of anti-inflammatory cytokines like IL-10, IL-13, IL-4, and TGF-β (Park et al., 2018). However, the TAMs within TME is considered as M2 type because of the immunosuppressive nature of TME. TAMs can be tackled by inhibiting the migration/infiltration of monocytes and their differentiation into M2 polarized form, by depleting TAMs from TME (Mehla and Singh, 2019) or by increasing the M1 effect *via* the polarization of M2 to M1 form. Immunostimulatory NPs mediated TME suppression by inhibiting M1 to M2 transition of macrophages is depicted in Figure 6 (II).

It has been observed that M1-derived exosomes and nanovesicles derived from those exosomes can polarize M2 macrophages to M1 type and trigger CD8^+^ T cell response, thereby reducing tumor progression (Choo et al., 2018). Besides, some other NPs like amino-functionalized and carboxyl-functionalized polystyrene NPs (Fuchs et al., 2016) and SPION (Zanganeh et al., 2016) can also suppress the M2 polarization.

Rodell et al. (2018) synthesized β-cyclodextrin NPs (CDNPs) loaded with R848 (dual TLR7/8 agonist) for efficient drug delivery into TAMs in order to promote polarization of M2 into tumoricidal M1 phenotype. Besides, when the immune checkpoint inhibitor anti-PD-1 antibody was used in combination with the drug loaded NPs, the immunotherapy response was further increased and tumor growth was controlled.

MDSCs (myeloid-derived suppressor cells) are another type of immune cells found in TME. They promote tumor progression, angiogenesis, metastasis, and release indoleamine 2,3-dioxygenase (IDO), NOS2, ARG1, TGF-β, and IL-10 leading to the activation of M2 macrophage and Treg, and suppression of T-cell proliferation (Park et al., 2018; Wang and Mooney, 2018). Thus, elimination of MDSCs can improve the suppression of TME and enhance cancer immunotherapeutic efficacy (Park et al., 2018). For this purpose, NPs were designed in such a way so that they can target and inhibit the functions of MDSCs. Immunostimulatory NPs mediated TME suppression by suppressing the MDSC cells is depicted in Figure 6 (III). For example, Plebanek et al. (2018) synthesized high density lipoprotein (HDL)-like NPs which interact with the scavenger receptor type-B1 present on MDSCs and exert antitumor effect by inhibiting MDSCs activity. As gemcitabine directly inhibits MDSCs, Sasso et al. (2016) constructed PEGylated lipid nanocapsules (LNCs) containing lauroyl-modified gemcitabine to suppress the MDSCs in TME and facilitate the T-cell proliferation. Besides, MDSCs production is also facilitated by cyclooxygenase-2 whose level is controlled by high-mobility group protein A1 (HMGA1). This protein also promotes tumor progression through the Wnt signaling pathway (Xian et al., 2017). Therefore, Wang et al. (2018) developed a liposome-protamine-hyaluronic acid (LPH) nanosystem loaded with HMGA1-siRNA and observed a significant increase in the number of T-cells and DCs with reduced number of MDSCs in a colon cancer model. Additionally, elevated expression of pro-inflammatory cytokines (IL-12a, IFN-γ, and TNF-α) and reduced expression of IL-10 and TGF-β was also observed (Wang et al., 2018).

IDO promotes tumor progression by degrading L-tryptophan (Trp) into L-kynurenine (Kyn) which stimulates Treg activation and MDSC infiltration (Holmgaard et al., 2015). Cheng et al. (2018) have synthesized a pH and MMP-2-responsive nanosized delivery platform for controlled delivery of NLG919 (IDO inhibitor) and ^D^PPA-1 (short D-peptide antagonist of programmed cell death-ligand 1). The simultaneous inhibition of Trp metabolism and immune checkpoint blockage stimulated T-cell activation and delayed tumor growth (Cheng et al., 2018).

The TME can also be suppressed by delivering immunomodulating agents that could improve the overall anti-cancer immune response (Park et al., 2018). We have already discussed about the various types of cells forming the TME and the possible ways to target them to improve cancer immunotherapy. Along with these cells, tumor cells secrete several cytokines and other factors that function as immunosuppressors which block the tumor infiltrating lymphocyte (TIL) activity. Therefore, targeting those tumor cell-mediated suppressive pathways can enhance the antitumor efficacy. For example, STAT3 signaling pathway promotes tumor growth via inducing hypoxia and angiogenesis, enhancing MMPs, and by promoting immunosuppressive cytokines secretion (Emeagi et al., 2013; Cui et al., 2016). Thus, delivery of STAT3-siRNA through NPs was shown to be effective in interfering with the STAT3 pathway and hindering the production of immunosuppressive cytokines (Jose et al., 2018; Nikkhoo et al., 2020). Similarly, TGF-β, an immunosuppressive cytokine pathway can also be targeted to suppress the TME. In a study, Schmid et al. (2017) encapsulated TGF-β inhibitors with NPs that were internalized to TME and results in the inhibition of tumor growth. Similarly, delivery of TGF-β-siRNA through NPs showed reduced number of Tregs and enhanced CD8^+^ T cell response (Xu et al., 2014).

## Cancer Theranostics Application of Immunomodulatory Nanoparticles

Apart from the delivery potential of antigens and adjuvants to APCs, few immunomodulatory NPs have also been used for diagnosis purpose. Theranostic NPs which are used for both diagnosis and therapeutic purposes offer a wonderful platform to revolutionize cancer therapy. Now-a-days, a number of molecular imaging techniques such as MRI, positron emission tomography (PET) imaging, X-ray computed tomography (CT), single-photon emission computed tomography (SPECT), and optical imaging are frequently used for diagnosis and therapy monitoring in cancer patients. The prerequisite for all these techniques is the accumulation of contrast agent at the target site. Besides, the multimodality imaging techniques such as the combination of SPECT/CT and PET/CT are widely used in most clinical diagnosis and ensures better revelation of functional and molecular information. On the other hand, MRI and CT are anatomical techniques which provide multidimensional structural, functional, and morphological information and are even superior to the SPECT/CT and PET/CT techniques due to their brilliant soft-tissue contrast resolution. Therefore, in this section we would like to discuss the potential applications of immunomodulatory NPs in molecular imaging.

Physical properties and size of the NPs are very important for their theranostic applications. Further, the theranostic NP system can be functionalized with specific ligands to target/bind tumor site for effective imaging. Various NPs are successfully used as CT and MRI contrast agents. For example, GNPs possess prolonged blood circulation time, delayed renal clearance, minimal cytotoxicity and very high x-ray attenuation, therefore it can be used as a promising contrast agent for CT imaging. Shanavas et al. (2018) synthesized gold-coated PLGA nanoshells and explored them as a contrast agent equivalent to that of iodine for CT imaging. Lin et al. (2017) also used unimolecular micelle-GNP hybrid as the CT imaging agent under both *in vitro* and *in vivo* conditions. They showed that treatment with the gold-nanohybrid significantly increased the CT contrast values as compared to Omnipaque *in vitro* as well as within the tumor in HepG2 xenograft mouse models. Besides, the gradual increase in CT values from 1 to 4 h after injection suggested the increased accumulation for nanohybrids within tumor *via* EPR effect. The gold-nanohybrid did not show any sign of systemic toxicity for at least 3 weeks post-injection. Moreover, GNPs and/or graphene oxide conjugated with Gd^3+^ could also be used as an MRI contrast agent as Gd^3+^ boosts the spin-lattice (T1) relaxation processes (Pitchaimani et al., 2017; Usman et al., 2018a,b). However, conjugation with GNP or graphene oxide further shortens the relaxivity of the MRI signals.

Iron oxide NPs (Fe_3_O_4_NPs) have widely been used as MRI contrast agents. Roy et al. (2015) synthesized a Fe_3_O_4_NPs-based carrier for drug delivery and visualized their tumor targeting ability *via* multimodality NIR/CT/MRI imaging techniques. Zhang et al. (2017) developed a magnetic nanocluster as aAPCs to stimulate and infiltrate CD8^+^ T cells into the tumor *via* magnetic control and monitored the process through MRI. Chen et al. (2010) developed magnetoplasmonic NPs by the combination of GNPs and Fe_3_O_4_NPs, and used them for cancer theranostics. The magnetoplasmonic NPs exhibited their applicability toward MRI in leukemia HL-60 cells by shortening the spin-spin (T2) relaxation time. Chen et al. (2016) designed magnetic-MSNs and demonstrated their utility as tumor-targeted MRI contrast agents. They further checked the effect of magnetic targeting on MRI and showed that tumor targeting and retention of the NPs was further increased under the influence of magnetic field, resulting in the appearance of much enhanced signal intensity in T2-weighted MR images within the tumor. Jing et al. (2019) constructed nanoflower composites of Fe_3_O_4_NPs core with MnO_2_ nanoshell for CT imaging *in vitro* in HeLa cells. Besides, the nanocomposites containing Mn^2+^ and Fe_3_O_4_ also exhibited *in vivo* T2/T1-weighted MRI response and a characteristic concentration dependent darkening and brightening effect of negative T2 and positive T1-MR contrast agent.

Several other researchers have also utilized GNPs and Fe_3_O_4_NPs in a single nanocomposite for multimodal imaging. For example, Bose et al. (2018) developed gold-iron oxide NPs coated with tumor-derived extracellular vesicles for T2-weighted MRI and indocyanine green -mediated NIR imaging in tumor bearing mice. Similarly, Nan et al. (2017), Ghorbani et al. (2018), and Zhong et al. (2019) also used gold and Fe_3_O_4_-derived nanocomposites for tumor targeted MRI.

## Conclusion and Future Perspective

Since last few decades, NP based immunotherapy has gone through expeditious development and are considered as a potential therapeutic strategy for cancer treatment. Here we have discussed about the role of NPs for successful cancer immunotherapy by activating APCs and T-cells, and targeting specific cells responsible for the immunosuppressive nature of the TME. Co-delivery of antigen and adjuvant to APCs enhances the cancer immunotherapeutic efficacy. NPs allow higher cellular uptake of immunostimulatory agents to induce T-cell and B-cell immune response, and sometimes behave as self-adjuvants.

Even though immunotherapy based on NPs has gained much interest for cancer treatment but the clinical translation of those immunostimulatory NPs is a major issue. Another important factor is the safety of immunostimulatory NPs as it has to interact with the immune system; therefore, the immunotoxicity should be assessed properly. Some NPs also alter the intracellular signaling pathway; so, this criterion should also be kept in mind while evaluating the toxicity of NPs. Sometimes, the immune system recognizes NPs as foreign material if they interact with the serum proteins, and an autoimmunity is generated against the NPs. Therefore, for successful clinical application, NPs must be designed in such a way so that they cannot induce hypersensitivity, allergic sensitization, or ROS, and can easily be cleared from the body.

These days most researchers working in the field of immunostimulatory NPs are trying to formulate nature-derived NPs as they are much safer and biocompatible than others, but they also require detailed investigation about the interactions with various biological components, including immune cells in the body. Overall, immunostimulatory NPs for cancer therapy is a promising research field. Although some limitations are there for its clinical application, but their therapeutic efficiency can be improved by doing more research on this field and designing safer nanocarriers to benefit the cancer patients.

## Author Contributions

NT, ST, SC, JD, and PS designed and wrote the manuscript, analyzed the data, designed the pictures, and critically reviewed the manuscript. NT and ST searched articles relating to the subject. All authors contributed to the article and approved the submitted version.

## Conflict of Interest

The authors declare that the research was conducted in the absence of any commercial or financial relationships that could be construed as a potential conflict of interest.

## Glossary

APCs, antigen presenting cells; aAPCs, artificial antigen presenting cells; ADCC, antibody-dependent cellular cytotoxicity; BMDCs, bone marrow-derived dendritic cells; BSA, bovine serum albumin; CCMV, cowpea chlorotic mottle virus; CDNPs, cyclodextrin nanoparticles; CHP, cholesterol-bearing pullulan; Clec9A, C type lectin receptors; CNTs, carbon nanotubes; CpG-ODNs, cytosine-phosphate-guanine oligodeoxynucleotides; CPMV, cowpea mosaic virus; DCs, dendritic cells; DC-Chol, 3β- (N- [N',N'-dimethyl aminoethane]-carbamoyl) cholesterol; DDA, dimethyl diocta decylammonium; DINP, dual immunotherapy nanoparticle; DMSA, dimercaptosuccinic acid; DOTAP, 1,2-dioleoyl-3-trimethylammonium-propane; ^D^PPA-1, short D-peptide antagonist of programmed cell death-ligand 1; ECM, extracellular matrix; EPR, enhanced permeability and retention; GDMON, glutathione depletion dendritic mesoporous organosilica nanoparticles; GDR, galactosyl dextran-retinal; GNPs, gold nanoparticles; GSH, glutathione; HDL, high density lipoprotein; HMGA1, high-mobility group protein A1; HPV 16, human papillomavirus 16; ICBT, immune checkpoint blockade therapy; IDO, indoleamine 2,3- dioxygenase; Kyn, kynurenine; LNC, lipid nanocapsules; LPH, liposome-protamine-hyaluronic acid; MAGE-A3, melanoma antigen gene family-A3; mDCs, myeloid dendritic cells; MDSCs, myeloid-derived suppressor cells; MHC, major histocompatibility complex; MIP 3α, macrophage inflammatory protein-3 alpha; MMP, matrix metalloproteinases; MRI, magnetic resonance imaging; MSN, mesoporous silica nanoparticle; 1MT, 1-methyl-DL-tryptophan; MWCNT, multiwalled carbon nanotube; NEs, nanoemulsions; NK cells, natural killer cells; NPs, nanoparticles; NY-ESO-1, new york esophageal squamous cell carcinoma-1; OVA, ovalbumin; PCL–PEG, polycaprolactone–polyethylene glycol; PCL-PEI, polycaprolactone-polyethylenimine; PD-1, programmed death-1; pDCs, plasmacytoid dendritic cells; PD-L1, programmed death ligand-1; PDT, Photodynamic therapy; PTT, Photothermal therapy; PEG, poly (ethylene glycol); PEG-PLA, poly(ethylene glycol)-block-poly(D,L-lactide); PEI, poly ethylenimine; PGA, poly (g-glutamic acid); PHMs, polymeric hybrid micelles; PLG, poly (D,L-lactide-co-glycolide); PLGA, poly (D, L-lactic-coglycolic acid); PLHMGA, poly (lactic-co-hydroxymethyl-glycolic acid); Poly (I:C), polyinosinic:polycytidylic acid; ROS, reactive oxygen species; siRNA, small interfering RNA; SLPs, synthetic long peptides; SPIONs, superparamagnetic iron oxide nanoparticles; TAAs, tumor-associated antigens; TAMs, tumor associated macrophages; TDLN, tumor draining lymph nodes; Th1 cells, T helper type 1 cells; TILs, tumor infiltrating lymphocytes; TLR, toll-like receptor; TMC, trimethyl chitosan; TME, tumor microenvironment; Tregs, regulatory T cells; Trp, tryptophan; TSAs, tumor specific antigens; TUSC 2, tumor suppressor candidate 2; VLPs, virus like particles; WNV, west Nile virus.
